# Development and Quality Control of a Population Pharmacokinetic Model Library for Caspofungin

**DOI:** 10.3390/pharmaceutics16060819

**Published:** 2024-06-17

**Authors:** Nuo Xu, Yufei Shi, Yixue Wang, Wenyao Mak, Wenyu Yang, Kar Weng Ng, Yue Wu, Zhijia Tang, Qingfeng He, Gangfeng Yan, Xiaoqiang Xiang, Xiao Zhu

**Affiliations:** 1 Department of Clinical Pharmacy and Pharmacy Administration, School of Pharmacy, Fudan University, Shanghai 201203, China; 22211030092@m.fudan.edu.cn (N.X.); yufei_shi@fudan.edu.cn (Y.S.); makwenyao@gmail.com (W.M.); 21211030109@m.fudan.edu.cn (W.Y.); zjtang@fudan.edu.cn (Z.T.); qf_he@fudan.edu.cn (Q.H.); 2 Hunan Key Laboratory for Bioanalysis of Complex Matrix Samples, Changsha 410000, China; 3 Department of Critical Care Medicine, Children’s Hospital of Fudan University, National Children’s Medical Center, Shanghai 200000, China; yixuewang08@aliyun.com (Y.W.); gangfeng_yan@fudan.edu.cn (G.Y.); 4 Department of Pharmacotherapy Services, Hospital Kuala Lumpur, Ministry of Health, Kuala Lumpur 50586, Malaysia; nkweng@moh.gov.my; 5 Department of Clinical Pharmacy, Shenzhen Children’s Hospital, Medical College, Shantou University, Shenzhen 518000, China; wuyue161@163.com

**Keywords:** caspofungin, individualized dosing, population pharmacokinetics, model library

## Abstract

Background: Caspofungin is an echinocandin antifungal agent commonly used as the first-line therapy for invasive candidiasis, salvage therapy for invasive aspergillosis, and empirical therapy for presumed fungal infections. Pharmacokinetic (PK) variabilities and suboptimal exposure have been reported for caspofungin, increasing the risk of insufficient efficacy. Objective: This work aimed to develop a caspofungin population pharmacokinetic (popPK) library and demonstrate its utility by assessing the probability of target attainment across diverse settings. Methods: We established a caspofungin popPK model library following a rigorous literature review, re-implementing selected models in R with rxode2. Quality control procedures included a comparison of different studies and assessing covariate impacts. Model libraries were primarily used to perform Monte Carlo simulations to estimate target attainment and guide personalized dosing in Candida infections. Results: A total of 13 models, one- or two-compartment models, were included. The most significant covariates were body size (weight and body surface area), liver function, and albumin level. The results show that children and adults showed considerable differences in pharmacokinetics. For *C. albicans* and *C. parapsilosis*, none of the populations achieved a PTA of ≥90% at their respective susceptible MIC values. In contrast, for *C. glabrata*, 70% of the adult studies reached a PTA of ≥90%, while all pediatric studies achieved the same PTA level. Conclusion: At the recommended dosage, adult patients showed notably lower exposure to caspofungin compared to pediatric patients. Considering body size, liver function, and serum albumin is crucial when determining caspofungin dosage regimens. Furthermore, further research is required to comprehensively understand the pharmacokinetics of caspofungin in pediatric patients.

## 1. Introduction

Echinocandins, including caspofungin, micafungin, and anidulafungin, represent a breakthrough in antifungal treatment. Caspofungin blocks the synthesis of an essential fungal cell wall component, β-(1,3)-D-glucan, leading to the osmotic instability and lysis of the fungal cell. Due to its selective action on fungal cells, caspofungin is usually well tolerated without common significant side effects. Caspofungin exerts potent activity against *Candida* and *Aspergillus* spp., serving as the first-line therapy for invasive fungal infections (IFIs), salvage therapy for invasive aspergillosis, and empirical treatment for presumed fungal infections in children over three months and in adults [1,2].

With poor oral bioavailability (<0.2%), caspofungin can only be administered by slow intravenous infusion over approximately one hour. About 97% of caspofungin binds to plasma proteins after entering the bloodstream. Following infusion, the plasma concentration of caspofungin declines in a polyphasic manner. The metabolism process is slow and occurs mainly via hydrolysis and N-acetylation [3].

Caspofungin’s favorable safety profile and relatively low potential for drug–drug interactions led to its widespread use in the prophylaxis and treatment of IFIs. However, reported pharmacokinetic (PK) variabilities and suboptimal exposure with standard dosing, especially in critically ill patients, increase the risk of insufficient efficacy [4,5]. Hence, personalized dosage recommendations and tailored adjustments, usually informed by measured observations and model-predicted exposure, are crucial to optimize treatment outcomes.

Caspofungin demonstrates a concentration-dependent inhibition of fungal growth [6]. The clinical efficacy of caspofungin in treating invasive aspergillosis relies on defined pharmacokinetic/pharmacodynamic (PK/PD) indices, like peak concentration/minimum effective concentration (C_max_/MEC), the area under concentration–time curve/minimum inhibitory concentration (AUC/MIC), and AUC/MEC [7,8]. Common targets include the ratio of the 24 h total drug AUC to MIC (AUC_0–24 h_/MIC) and the ratio of the 24 h free drug AUC to MIC (*f*AUC_0–24 h_/MIC) [9,10]. AUC_0–24 h_/MIC, the most influential PK/PD index for caspofungin, exhibits substantial variability among intensive care unit (ICU) patients [11,12]. Despite preclinical support, these targets have yet to be confirmed and validated in clinical studies, posing challenges in their application to clinical practice.

Population pharmacokinetic (popPK) models describe the PK profiles of the studied population and can evaluate the effect of various covariates on PK variabilities. When integrated with Bayesian forecasting, which combines prior knowledge with new observational data, this approach has become increasingly utilized for designing and adjusting dosing regimens in clinical practice [13]. The Bayesian method requires a well-defined structural model that includes parameters informed by prior distributions and is continually updated with new clinical observations, ensuring that dosing regimens are tailored to the specific needs and responses of patients. Despite numerous PK/PD studies on caspofungin, existing model information has not been quantitatively integrated to guide further research. Therefore, a model library would be invaluable for facilitating model-informed precision dosing (MIPD) strategies by compiling relevant data.

This study aimed to develop a caspofungin population pharmacokinetic (popPK) library and demonstrate its utility by assessing the probability of target attainment across diverse settings. This consisted of three specific objectives: (1) to identify and re-implement existing popPK models using an open-source R package; (2) to perform a quality control of the developed model library by comparing PK metrics and evaluating the impact of covariates; and (3) to demonstrate the utility of the model library by assessing the probability of target attainment (PTA) across diverse study settings.

## 2. Materials and Methods

### 2.1. Development of Model Library

#### 2.1.1. Search Strategy

Four databases, including PubMed, Embase, Web of Science, and Scopus, were searched for popPK model studies of caspofungin published before 25 November 2022, following the Preferred Reporting Items for Systematic Reviews and Meta-Analyses (PRISMA) guidelines [14]. The search strategy encompassed keywords pertinent to the focal medication (‘Caspofungin’ or ‘Cancidas’ or ‘MK 0991’ or ‘L 743,872’), along with terms specifically denoting popPK modeling approaches, such as population pharmacokinetic, NONMEM, MONOLIX, or nlmixr. Additionally, all reference lists from selected articles were searched to ensure the comprehensiveness of our study. Two independent authors conducted the literature research, and another two senior investigators performed the data validation [14]. The comprehensive search strategies employed for each database, as well as the detailed inclusion and exclusion criteria utilized in the study, can be found in the Supplementary Materials.

All studies identified from the databases and other sources were screened to evaluate their eligibility based on the following consolidated criteria: (1) the subject of the studies was human, including healthy volunteers and patients; (2) caspofungin was the study drug; (3) popPK or PK/PD analysis was conducted in the study; and (4) the study was published in English. A publication was excluded if (1) it was not an article or only focused on the methodology, algorithms, or software comparisons or (2) critical PK parameters were insufficient.

#### 2.1.2. Literature Evaluation

Literature evaluations were implemented to diligently identify and resolve any anomalies or inconsistencies pertinent to the construction of the model library [15]. The quality of the popPK study was evaluated based on a checklist with 33 items adapted from the guidelines (see Supplementary Materials) [16,17]. The literature evaluation was divided into five parts: title and abstract, introduction, methods, results, and discussion and conclusion. A risk of bias assessment was conducted. Each item received one point if the study information met the criteria; otherwise, zero points were assigned. Compliance, reflecting popPK study quality, was calculated as compliance% = (items reported/total items) × 100%. The risk of bias plot was conducted using the “ggplot2” package (version 3.3.5; https://ggplot2.tidyverse.org, accessed on 5 November 2022) in R software (version 4.1.1; http://www.r-project.org, accessed on 5 November 2022).

#### 2.1.3. Data Extraction

A standardized data extraction method was systematically employed to facilitate data collection from all eligible studies. This process encompassed the following key aspects: (1) demographic characteristics (e.g., race, age, sex, and weight range); (2) the study design (e.g., type of study, number of subjects and observations, dosage regimens, administration, sampling schedule, and detection method) [16]; (3) popPK modeling characteristics (e.g., software/algorithm, final PK parameters, model validation); (4) investigated and identified covariates information in popPK models; (5) PK/PD targets used for simulation if applicable; and (6) model application and recommended dosage regimens.

#### 2.1.4. Model Re-Implementation

After extracting all relevant information from the models, we re-implemented them using the open-source R package “rxode2” package (version 2.0.12; https://nlmixr2.github.io/rxode2/index.html, accessed on 8 December 2022), with all parameters set to the final parameter estimates reported in the literature. All of the model codes are provided in the Supplementary Materials.

### 2.2. Quality Control of Model Library

#### 2.2.1. Comparison of Studies

Quality control (QC) measures were implemented to screen and resolve any discrepancies or issues arising during the establishment of the model library, ensuring rigorous standards were maintained. We constructed three age-stratified virtual patient cohorts (comprising infants, children, and adults), thoughtfully designed to accurately mirror the specific target population of their corresponding models.

Virtual populations were created and divided into three age groups: infants (10 kg, 75 cm, 1 year old), children (20 kg, 100 cm, 6 years old), and adults (70 kg, 40 years old). Infants and children adopted the standard dosage regimen, 70 mg/m^2^ on the first day, followed by 50 mg/m^2^ once daily, whereas adults followed a different dosage regimen: 70 mg on the first day, followed by 50 mg once daily. Caspofungin was administered intravenously over one hour to all groups once daily. The Mosteller formula [18] was used to calculate body surface area (BSA) values for infants and children, which were set at 0.441 m^2^ and 0.79 m^2^, respectively. All patients reached a steady state with the labeled dosing regimen. Concentration–time profiles were simulated with the “rxode2” package (version 2.0.12; https://nlmixr2.github.io/rxode2/index.html) in R.

We conducted a similarity comparison assessment to ensure the accuracy of constructing the developed model library. The idea behind this assessment is straightforward. If the models have been accurately implemented, the concentration–time curves for the models with similar target populations should be comparable as they describe the PK behavior of the same drug in the same population. The model performance is considered comparable if its typical maximum concentration (C_max_) falls within the range of 50–200% of the median C_max_ across all studies (see Supplementary Materials).

#### 2.2.2. Assessment of Covariates Impact

Clearance (CL) is a crucial parameter for AUC, while the apparent volume of distribution (V_d_) is an important PK parameter for C_max_. AUC and C_max_ play central roles in the individualized dosing of caspofungin. Thus, comparing the effects of different covariates on CL and V_d_ was necessary. The effect of covariates was presented through a forest plot using the “ggplot2” package (version 2.0.0; Tidyverse packages) in R. For continuous covariates, the maximum and minimum values from the included studies were extracted and used for calculating the range of the effect of different covariates on CL or V_d_. For covariates included in multiple studies, a uniform range was set up for the comparison based on the demographic information in the included studies. For binary covariates, such as disease situation. The uncommon condition (critically ill disease) was treated as the reference (COV_i_ = 0), while the uncommon condition (not critically ill disease) was treated as the test (COV_i_ = 1). CL_i_ = CL_common_ + CL_diff_ × COV_i_. The range of CL_i_ was [CL_common_, CL_common_ + CL_diff_] (if CL_diff_ > 0) or [CL_common_ + CL_diff_, CL_common_] (if CL_diff_ < 0). Then, the values of CL and Vd were further normalized by the reference to the median covariate value (equation). We regarded covariate effects beyond the 80–125% range as clinically significant, in accordance with the standard employed in bioequivalence studies [19].

$$The\;effect\;value\;of\;covariates=\frac{Upper\;or\;lower\;limit}{Reference\;value}\;*100\%$$

### 2.3. Application of PopPK Model Library

Louie et al. reported comparable Candida albicans reductions in kidney tissue and proposed AUC_0–24 h_/MIC as the optimal PK/PD index for caspofungin [20]. Based on the in vivo PK/PD studies, the PK/PD targets for *C. albicans*, *C. glabrata*, and *C. parapsilosis* were 865, 450, and 1185, respectively [10,21]. According to the clinical and laboratory standards institute (CLSI) MIC breakpoint for the in vitro broth dilution susceptibility testing of *Candida* spp., MICs for each Candida species were selected (*C. albicans*, susceptible ≤ 0.25 mg/L; *C. glabrata*, susceptible ≤ 0.12 mg/L; *C. parapsilosis*, susceptible ≤ 2 mg/L) [22]. After the popPK model library of caspofungin was established, a Monto Carlo simulation was conducted to predict the probability of caspofungin reaching the PK/PD target under labeled dosing regimens and specific MIC. The steps were as follows: (1) simulate PK profiles of caspofungin; (2) calculate the steady-state AUC_0–24 h_ using the trapezoidal method; and (3) calculate the PTA under specific MIC settings.

## 3. Results

### 3.1. Overview of Included PopPK Studies for Caspofungin

#### 3.1.1. Identification of Included Studies

**Figure 1:**
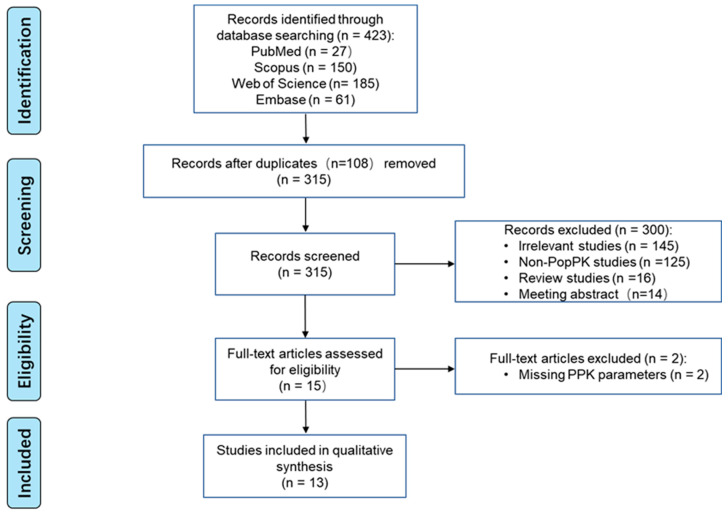
PRISMA flow diagram for the identification of caspofungin population pharmacokinetics (PPK) studies.

Figure 1 presents the PRISMA flowchart for study identification. From PubMed, Scopus, Web of Science, and Embase, 26, 150, 185, and 61 studies were retrieved, respectively, with no additional records from other sources. A total of 13 studies were included in our model library for subsequent analysis.

#### 3.1.2. Evaluation of Literature

**Figure 2:**
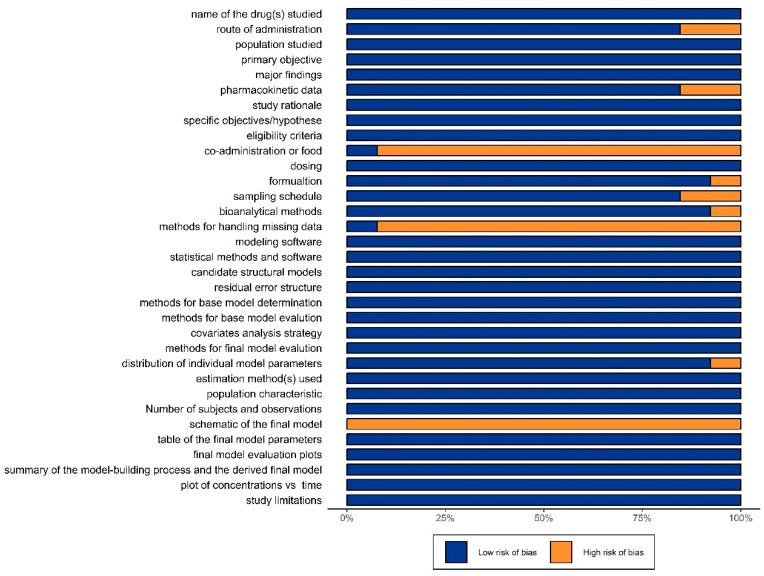
Evaluation of the included population pharmacokinetics studies (the risk bias plot).

The risk map concerning the bias of the literature is summarized in Figure 2. Two studies lacked a description of PK data in their background sections, and two did not include the sampling schedule. One study did not mention the drug formulation, bioanalytical methods, and the distribution of individual model parameters. None of the included studies detailed the methods of handling missing data, and fewer than 20% of studies reported a specific way of handling the data below the quantification limit. Nevertheless, all studies achieved a compliance rate of 85%, indicating that all studies are of good quality.

#### 3.1.3. Study and PopPK Model Characteristics

The characteristics of each study are summarized in Table 1. All included studies were published from 2011 to 2022, with nine [23,24,25,26,27,28,29,30,31] being clinical trials and four [11,12,32,33] being observational studies. The total number of participants ranged from 12 to 299 (IQR (19, 48)). Wang et al. [30] and Wu et al. [29] enrolled both patients and healthy subjects, while the others only enrolled invasive candidiasis patients. Three studies enrolled a pediatric population between three months and 18 years, while the rest included adults only. Five studies focused on transplant patients, including allogeneic hematopoietic stem cell transplant [25,32], heart transplant [29], lung transplant [30], and liver transplant [26]. While most studies collected only plasma concentrations, Pressiat et al. [26] collected plasma and peritoneal fluid samples. Sparse sampling strategies were adopted in all three pediatric and two adult studies [23,34], with various bioassay methods used to determine caspofungin concentrations. Despite methodological differences, steady-state plasma concentrations under standard dosing regimens remained above the lowest limits of quantitation (LLOQ, range [0.084–0.6 mg/L]), suggesting their minimal influence on the results.

The modeling strategies and final PK parameters of the included studies are summarized in Table 2. Ten studies [11,12,23,27,28,29,30,32,33,34] used NONMEM software, with first-order conditional estimation with interaction (FOCE-i) being the most used algorithm [11,12,23,27,28,32,33,34]. Most studies employed a two-compartment model to describe caspofungin PKs, although two studies [25,34] utilized a one-compartment model due to sparse sampling schedules that may not accurately represent the characteristics of a two-compartment PK model.

All studies incorporated between-subject variability (BSV), with a median of 27.5% (range: 11.8–42.3%) in CL across the 13 studies. Residual unexplained variability was described by proportional [11,23,25,26,27,28,31,32,33,34], additive [12,30], or combined models [29]. Proportional errors with coefficients of variation ranged from 12.2% to 36% in eleven studies, while three studies [12,29,30] included addictive errors ranging from 0.0941 to 0.73 mg/L. An integration of inter-occasion variability on CL was estimated to be 17.2% and 16.0% in studies by Gastine et al. [33] and Würthwein et al. [28], respectively.

All included popPK models conducted internal validation, including goodness-of-fit tests, a visual predictive check, a predicted–corrected visual predictive check, and normalized prediction distribution error plots. Additionally, ten [11,12,25,27,28,29,30,32,33,34] out of thirteen studies presented results of bootstrap analyses. Würthwein et al. [28] used external validation to further verify their model.

Ten studies [11,12,23,25,26,27,29,31,33,34] performed Monte Carlo simulations to evaluate existing dosing regimens or optimize caspofungin therapy, comparing different dosage regimens or evaluating caspofungin PKs and drug interaction in specific patient populations. In nine [11,12,23,25,26,29,31,33,34] of these studies, the simulation targeted AUC_0–24 h_/MIC as the PK/PD. Pressiat et al. [26] concluded that the standard caspofungin dose was sufficient, while five studies [11,12,23,29,34] explicitly advocated for adjusting dosing regimens based on covariates, suggesting higher doses (70–150 mg) considering factors like disease condition, albumin (ALB) levels [29], fungal colony type, and liver function.

Niu et al. [25] concluded that the current recommended dose of caspofungin was sufficient for pediatric patients and did not require higher doses. Gastine et al. [33] reported that a twice-weekly extended dosing regimen of 200 mg/m^2^, with a maximal total dose of 200 mg, should yield comparable average weekly exposures to the approved daily-dosing regimen. Additionally, Yang et al. [27] included BSA as a significant covariate on both CL and V_d_, supporting the adoption of a BSA-based dosing regimen.

### 3.2. Overview of PopPK Model Library

#### 3.2.1. Comparison of Caspofungin PK Profiles

**Figure 3:**
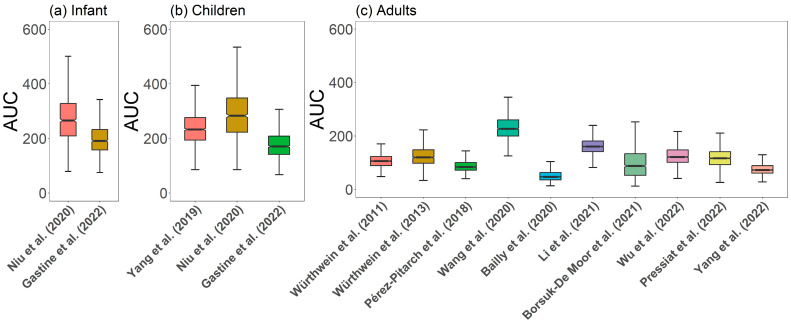
Caspofungin main pharmacokinetic parameter (AUC) at steady state of (**a**) infants, (**b**) children, and (**c**) adults. Virtually all patients were assumed to be male and received caspofungin at a dose of 70 mg on the first day, followed by 50 mg once a day as an infusion for 1 h for adults and 70 mg/m^2^ on the first day, followed by 50 mg/m^2^ once a day as an infusion for 1 h for infants and children [11,12,25,26,27,28,29,30,31,32,33,34].

Simulated caspofungin concentration–time profiles are displayed in Figure S1. A comprehensive comparison of all simulated PK parameters for caspofungin is shown in Figure 3. The median C_max_ values of children (23.56 mg/L) and infants (25.65 mg/L) were higher than that of adults (8.75 mg/L), likely due to the lower V_d_ in children and infants (adults vs. pediatric population: [2.21–9.01 L] vs. [1.36–2.21 L]). Moreover, the pediatric population demonstrated a higher CL per kilogram than adults, with weight-normalized median CL values of 0.0083 L/h/kg (range: 0.005–0.011 L/h/kg) and 0.0086 L/h/kg (range: 0.006–0.011 L/h/kg) for children and infants, respectively, compared to 0.0061 L/h/kg (range: 0.003–0.014 L/h/kg) in adults.

In the pediatric population, three studies [25,27,33] displayed similar PK characteristics in both infants and children. Among adult patients, Wang et al. [30] reported higher AUC and C_max_ than other studies under the same dosage regimen, while Bailly et al. [31] showed much lower exposure. However, the remaining eight studies showed similar PK profiles despite variations in patients’ disease conditions (Figure S1). The subjects in Wang et al.’s study [30] were lung transplantation recipients receiving ICU follow-up treatment, exhibiting lower CL and V_d_.

#### 3.2.2. Covariate Screening and Influence Analysis

**Figure 4:**
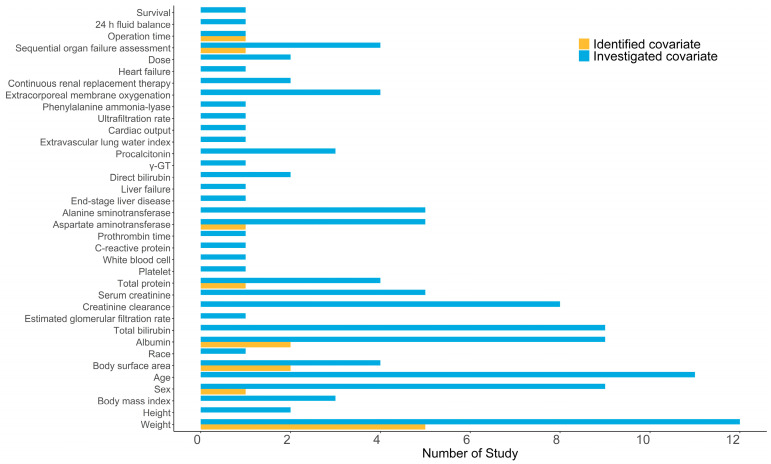
A histogram of the amount of investigated and identified covariates in included studies.

All tested covariates that affected CL, V_d_, intercompartment clearance (Q), and the distribution volume of the peripheral compartment (V_p_) are summarized in Table 3. The stepwise covariate screening included forward inclusion and backward elimination. A comparison of identified and investigated covariates is presented in Figure 4.

Four studies [11,26,31,32] did not include any covariates, likely contributing to the high consistency of the included patients. The most influential covariate was body size, such as body weight and BSA. Three [15,19,34] studies (23.1%) found body weight closely related to CL, and five studies [12,23,28,33,34] (38.5%) indicated its impact on V_d_. Yang et al. [27] and Niu et al. [25] identified BSA as a crucial covariate that affects both CL and V_d_. Moreover, three studies [23,29,34] confirmed ALB level as a significant covariate affecting CL. In their final model, Niu et al. [26] and Li et al. [24] included the liver function biomarker as a covariate. Interestingly, among the studies including pediatric populations, two [25,27] identified BSA as a significant covariate on CL and V_d_, supporting dosing based on BSA. Würthwein et al. [28] found that the PK of caspofungin was not altered by the coadministration of liposomal amphotericin B. Three studies [11,29,30] investigating the difference in PK characteristics in patient groups who had received continuous renal replacement therapy (CRRT) or extracorporeal membrane oxygenation (ECMO) concluded that these factors had no significant effect.

**Figure 5:**
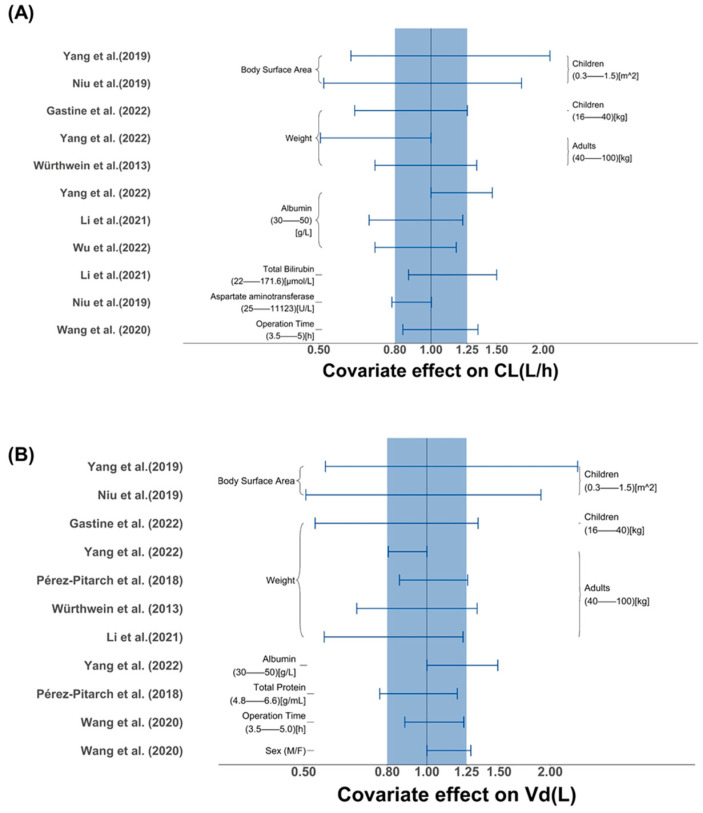
(**A**) The effect of covariates on CL (L/h) of caspofungin in included studies. (**B**) The effect of covariates on V_d_ (L) of caspofungin in included studies. The horizontal bars represent the covariate effect on clearance in each study. The typical value of clearance in each study was considered to be 1. The effect of each covariate for clearance is displayed by the ratio of clearance in the range of each covariate to the typical clearance value. The shaded area ranges from 0.8 to 1.25 [23,25,26,27,28,29,30,33,34].

Identified covariates on CL and V_d_ were visualized using the forest map in Figure 5. Figure 5A highlights BSA as a pivotal determinant of CL in children, with weight significantly influencing CL in adult and pediatric populations. The impact of body size measures exceeds the conventional range (80–125%), suggesting considerable CL variability. Despite variations in covariates across studies due to different populations, the profound influence of body size on caspofungin CL remains consistent. Compared to the reference value, six [12,23,25,27,29,33] out of the seven studies demonstrated a significant impact of body size on V_d_ with changes greater than 20% under the normal range of body size. Additionally, two studies [12,27] showed the influence of ALB to exceed a 20% change in V_d_ within the normal range. Wang et al. found that male gender was associated with increased caspofungin V_d_ (Figure 5B). Li et al. [23] and Niu et al. [25] showed liver function to have changes over 20% under a wide range of liver functions. Three studies [23,29,34] included ALB as a significant covariate affecting CL, with changes exceeding 20% within the normal range of the ALB level.

### 3.3. Model Library Applications

**Figure 6:**
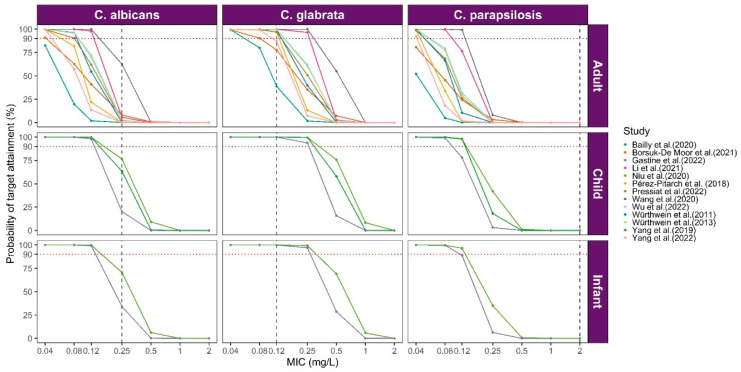
The probability target achievement of caspofungin in included studies. The red dashed line represents a 90% target attainment rate, while the three vertically oriented dashed lines of different colors correspond to the respective clinical breakpoints for MICs of each fungus [11,12,25,26,27,28,29,30,31,32,33,34].

In our model library, 77% of studies incorporated AUC/MIC as the PK/PD target for simulation. The simulated PTA for each published popPK model was assessed with three Candida strains, the results of which are presented in Figure 6. For *C. albicans* (MIC = 0.25 mg/L) and *C. parapsilosis* (MIC = 2 mg/L), none of the populations achieved a PTA of ≥90%. However, for *C. glabrata* (MIC = 0.12 mg/L), 70% of studies focused on adults reached a PTA of ≥90%, while all pediatric patients achieved a PTA of ≥90%. The PTA results suggested that caspofungin may be underexposed in adult patients, indicating the necessity of basing drug dosage on the Candida strains’ results and the corresponding MIC value.

## 4. Discussion

Numerous PK studies on caspofungin have been conducted recently, with several popPK studies aiming to explain PK variability. To our knowledge, this study was the first to build and share a parametric popPK model library of caspofungin. The library is characterized by simulations of concentration–time profiles and covariate effect assessments, where we demonstrated the potential of the constituent models in estimating the AUC, C_max_, and PTA of caspofungin. This work provides evidence for advocating individualized dosing not only based on body size but also potential covariates such as liver function and hypoalbuminemia.

### 4.1. Pediatric Patients

The PKs of caspofungin vary substantially among pediatric patients. All three pediatric studies within the model library consistently applied allometric scaling, aligning with the standard pediatric dosing approach that utilizes body surface area as the basis for medication administration. Children showed higher CL (L/h/kg) than adults per kilogram, possibly due to differences in distribution rates into hepatic tissue. In vitro data suggested the involvement of uptake transporters, such as OATP1B1, in caspofungin tissue distribution, particularly in hepatic uptake [35]. Studies [24,36,37] suggested that factors like accelerated liver uptake, larger liver-to-body ratios, and increased hepatic blood flow contribute to heightened caspofungin CL in infants and toddlers. However, conflicting findings exist regarding age-dependent differences in hepatic OATP1B1 expression and its impact on caspofungin uptake. Thomson et al. reported no age-dependent differences in hepatic OATP1B1 expression across pediatric groups. Sáez-Llorens et al. [38] suggested that caspofungin CL increases from infancy to childhood and then decreases through adolescence into adulthood. Pediatric patients generally exhibit higher CL (L/h) and V_d_ (L) compared to adults under standard dosing regimens, attributed to factors such as reduced blood flow, higher body fat-to-lean mass ratios, and decreased total body water. These physiological and pathological distinctions, particularly pronounced in infants, can significantly influence caspofungin’s absorption, distribution, metabolism, and excretion.

Findings from included studies indicated that adjusting the caspofungin daily dose based on BSA, rather than weight, might be more reasonable in pediatric patients. Three studies [25,27,33] focused on children with a median age of around six years used body size for allometric scaling; one included weight, and two studies included BSA. Gastine et al. (48 subjects) estimated weight-normalized CL and V_d_, implicitly assuming the power of 1 for the impact of weight on CL and V_d_. Niu et al. (48 subjects, 139 observations) showed that the power of BSA on CL was estimated to be 0.89 (RSE 11.36%), and the power of BSA on V_d_ was fixed to 1, respectively. In Yang et al. (48 subjects, 159 observations), the power of BSA on CL and V_d_ was estimated to be 1.3 (RSE 13.8%) and 1.5 (RSE 13.5%). Overall, the body size metrics have positive correlations with CL and V_d_. Due to the under-power design of the study [39], the slight deviations in the estimated exponents from the theoretical allometric relationship should be interpreted with caution.

Sáez-Llorens et al. [38] found that a BSA-based dosage of 25 mg/m^2^ QD in neonates under three months achieved plasma concentration profiles similar to adults receiving a standard 50 mg daily dose [38]. However, PK variability was higher in pediatric patients with a lower body weight, necessitating individualized dose adjustments. Beyond BSA-based dosing, considering blood biochemical parameters and physiological conditions, such as serum ALB level, hepatic function, and blood flow, is crucial. Caspofungin is occasionally used in very low-birth-weight infants, but PK data for this group are scarce [40]. This scarcity emphasizes the necessity for large-scale, prospective trials to establish caspofungin’s efficacy, safety, optimal dosage, and role in neonatal candidiasis, particularly in infants under three months with a very low birth weight.

### 4.2. Adult Patients

Insufficient drug exposure is common with the labeled dosing regimen in adult patients. In this model library, five of the ten adult studies proposed increasing the dosage of caspofungin to achieve target exposure levels. Caspofungin dosing differences exist between adults and children. Lower adult dosages correspond to lower drug concentrations over time compared to children (Figure S1), indicating a need for dosing strategy improvement. In patients with normal liver and kidney function, hypoalbuminemia can result in elevated levels of unbound drugs, potentially leading to an increased CL, especially in highly protein-bound antibiotics like caspofungin. Additionally, as a hydrophilic drug, caspofungin’s V_d_ may increase due to fluid shifts and extensive fluid resuscitation [41,42].

Critically ill patients often present with concomitant conditions such as obesity, hepatic impairment, and specific infusion regimens, contributing to the high BSV of caspofungin PKs [43]. Treatment modalities like ECMO and CRRT are frequently employed in ICU settings. Studies have yielded conflicting results regarding the impact of ECMO on caspofungin CL, with one study [30] suggesting an increase and another [31] finding no discernible effect, likely due to caspofungin’s high solubility in water and methanol. Critically ill patients exhibit lower caspofungin exposure compared to healthy subjects [5]. Betts et al. supported a wide safety margin for caspofungin, allowing for higher-dose therapy up to 150 mg daily if clinically indicated [44]. However, arguments against reducing caspofungin doses in critically ill patients with hypoalbuminemia and abnormal liver function suggest that a uniform, flat dosing strategy may not be optimal [45]. Consensus leans towards the importance of therapeutic drug monitoring in critically ill patients with hypoalbuminemia, with or without abnormal liver function, or in cases where strains with elevated minimum inhibitory concentrations need to be addressed.

Borsuk-De Moor et al. [11] noted a time-varying CL of caspofungin in ICU patients: 0.563 L/h on day 1, 0.737 L/h on day 2, and 1.01 L/h on day 3, respectively. This escalating CL may decrease AUC and impair efficacy, but few models considered time-dependent variables. The extended monitoring of caspofungin therapy is recommended for personalized medication optimization.

### 4.3. Towards Precision Dosing

The model library of caspofungin popPK models developed in this study has the potential to accelerate the implementation of MIPD. Leveraging our model library, researchers could rapidly perform external validations on their data, efficiently selecting the most appropriate model for local use, thereby speeding up individualized dosing [46]. Integration with the “PopED” package further enhances its utility by allowing the selection of published library models as prior information for clinical trial design.

However, translating preclinical PK/PD targets into clinical benchmarks remains challenging. Investigations into the MIC of caspofungin are notably limited, chiefly attributable to the challenges in cultivating fungus, the extended duration of experimental procedures, and an inadequate favorable return on investment. Notably, a recurring theme across studies is the lack of well-established clinical PK/PD targets, leading to reliance on preclinical counterparts. CLSI guidelines specify MIC breakpoints for *Candida* spp. in vitro broth dilution tests, but significant differences in MICs among bacterial species underscore the importance of bacterial culture in clinical practice. In our model library about adults, PK/PD cut-off values vary for *C. albicans*, *C. glabrata*, and *C. parapsilosis*. These findings suggest significant variability in establishing caspofungin cut-off values across populations, posing challenges for clinicians and researchers in determining optimal dosing strategies. For caspofungin, the current target values are derived from preclinical studies and are in urgent need of confirmation through clinical research.

Disparities in PK profiles between pediatric and adult populations highlight the need for further research. The pediatric and adult populations exhibit marked disparities in PK profiles, particularly with much higher caspofungin exposure levels observed in children compared to adults, up to 1.5 times greater with standard dosing regimens. This discrepancy may stem from two potential reasons: differences in the PK/PD relationship between children and adults and the need to optimize the current dosing regimen, suggesting either dose increases for adults or decreases for children. However, due to limited research in pediatric populations, further PK/PD studies in this population are essential to elucidate the underlying causes.

Additionally, assessing the concentration of the free drug is essential, as only the unbound form exerts therapeutic effects, though this aspect is often overlooked in studies of caspofungin. Reduced plasma protein levels in vitro were found to increase the antifungal activity of caspofungin and lower the pharmacodynamic target AUC_total_/MIC, likely by increasing the free concentration [47]. However, none of the included studies measured free caspofungin concentrations. In cases where fAUC_0–24 h_/MIC was employed as the PK/PD target, it was calculated based on the total AUC/MIC target and caspofungin’s plasma protein binding rate of 97%.

### 4.4. Limitations

Our research has certain limitations. Firstly, due to the limited number of studies involving pediatric patients, we were unable to comprehensively investigate and compare the PK of children and infants. Furthermore, as this model library focused on summarizing significant covariates influencing caspofungin PKs and comparing popPK modeling across different age groups, we only replicated the published models to align with our study objectives and the characteristics of the medications under investigation. Additionally, PK/PD targets for PTA calculation were derived from in vitro data provided by CLSI, which may lead to discrepancies in PTA accuracy due to the absence of in vivo studies in humans. Finally, our study was restricted to the English-language literature, potentially omitting relevant studies published in other languages. 

## 5. Conclusions

The model library of parametric population pharmacokinetic models for caspofungin is helpful in promoting model-informed precise dosing. The optimization of the caspofungin dosing regimen should consider the patient’s liver function and hypoproteinemia. Additionally, prospective PK/PD studies of caspofungin in pediatrics are warranted to elucidate the exposure–response relationship associated with this medication.

## Figures and Tables

**Table 1:** Characteristics of included population pharmacokinetics studies.

Study (Publication Year)	Country (Type of Study)	Number of Subjects (Male/Female)	Basic Entry Standard	Number of Observations	Sampling Schedule	Age (Years) Mean ± SD Median [Range]	Weight (kg) Mean ± SD Median [Range]	Daily Dose Mean ± SD Median [Range]	Bioassay [LOQ]
Würthwein et al. (2011) [32]	Germany (clinical trial)	19 (11/8)	Adults allo-HSCT recipients, immunocompromized	239	Day 1 and Day 4: immediately before administration; 0.5 to 1.5 h, 1.5 to 3 h, 3 to 5 h, 5 to 11 h, and 22 to 23 h after administration; Thereafter: random time points twice weekly until the end of treatment	43.4 [20.1–57.6]	71.2 [56–99.2]	70 mg QD on day 1, followed by 50 mg QD, IV infusion over 60 min	HPLC [0.15 mg/L]
Würthwein et al. (2013) [28]	Germany (clinical trial)	46 (21/25)	Adults with invasive Aspergillosis, immunocompro-mized	468	day 1 (immediately prior to dosing and 2 h [peak level], 3 h, 5 to 7 h, and 24 h [trough level] after the start of infusion); peak and trough time points on days 4, 7, 14, 28 h	61 [18–74]	76 [43–104]	70 mg, QD; 100 mg, QD; 150 mg, QD; 200 mg, QD, IV infusion over 120 min	LC-MS/MS [0.084 mg/L]
Pérez-Pitarch et al. (2018) [12]	Spain (clinical trial)	12 (6/6)	Critically ill adults on CVVHD	105	Day 3 and later: predose, 0.5, 1, 1.5, 2, 2.5, 3, 5, 7, 9, 24 h after the start of infusion	73 [56–78]	75 [60–88]	NR	HPLC [NR]
Yang et al. (2019) [27]	France (clinical trial)	48 (28/20)	Children in ICU	159	NR	6.07 ± 2.74 5.09 [2.05–11.77]	22.78 ± 8.71 21 [11.8–47.5]	70 mg/m^2^ (loading dose on day 1), 50 mg/m^2^ QD.	HPLC/MS [0.25 mg/L]
Wang et al. (2020) [30]	China (clinical trial)	ECMO group 12 (9/3)	Adults on ECMO after LT	271	predose, 0.5, 1, 2, 4, 8, 12, 24 h after the start of the infusion	65 [60–67]	64 [59–69.3]	50 mg QD	UPLC-MS/MS [0.39 mg/L]
		Control group 7 (5/2)	Adult patients never on ECMO after LT			59 [56–62]	65 [53–65]		
Bailly et al. (2020) [31]	France (observational study)	13 (10/3)	Adult Patients in ICU with proven or suspected invasive candidiasis	NR	0, 2, 3, 5, 7, 24 h postinfusion	53 [34–55]	76.5 [60–85]	50 mg QD with a 140 mg loading dose, IV infusion over 60 min	LC-MS/MS [0.5 mg/L]
Niu et al. (2020) [25]	China (clinical trial)	48 (31/17)	Children with allo-HSCT	139	an opportunistic sampling strategy	6.58 ± 3.7 [0.61–14]	21.7 ± 10.3 [7.5–54]	loading dose of 70 mg/m^2^ followed by 50 mg/m^2^	HPLC [0.6 mg/L]
Borsuk-De Moor et al. (2021) [11]	Poland (observational study)	30 (16/14)	ICU patients	180	0.5, 2, 4, 8, 12, 24 h	53 [28–76]	74 [40–150]	70 mg intravenously on the first day and at 50 mg i.v on the consecutive days	HPLC [0.18 μg/mL]
Li et al. (2021) [23]	China (observational study)	42 (31/11)	ICU patients with IFIs	140	1,3,6, 24 h on Day 4	56.82 ± 16.39 [20–88]	59.18 ± 11.40 [41–84.5]	a 70 mg loading dose and a 50 mg maintenance dose	LC-MS/MS [0.2 μg/mL]
Gastine et al. (2022) [33]	Germany (clinical trial)	48 (26/22)	Children aged 3–17	NR	Day 1, Day 4 and Day 9	6 [0–16]	21.5 [9.4–79.5]	CAS I: 1 mg/kg	NR
								CAS II: 50 mg/m^2^	
								CAS III: 70 mg/m^2^	
								CAS IV: 50 mg/m^2^	
Wu et al. (2022) [29]	China (clinical trial)	HTx group 27 (22/5)	27 HTx	414	predose, 1, 2, 6, 10, 16, 24 h	HTx group 50 [20–73]	HTx group 59.5 [43.5–76]	1 h IV infusion at a dose of 50 mg QD after a loading dose of 70 mg	LC-MS/MS [0.4 mg/L]
		non-HTx group 31 (21/10)	31 non-HTx			Control group 58 [22–78]	Control group 62 [48–100]		
Pressiat et al. (2022) [26]	France (clinical trial)	20 (9/11)	Adult LT recipients admitted to the liver ICU	395 plasma and 50 PF samples	predose, 1, 2, 4, 8, 12, 24 h D1, D3, D8	45 [40.7–50]	72 [62–81]	A loading dose of 70 mg and then 50 mg per day (or 70 mg per day if the recipient > 80 kg), IV infusion over 1 h	HPLC [0.5 mg/L]
Yang et al. (2022) [34]	China (observational study)	299 (207/92)	Patients who have been diagnosed with confirmed or probable candidiasis	921 plasma samples	C_min_ samples at interval windows of 22–24 h post-dose, other samples at interval windows of 0–12 h and 12–24 h post-dose	44 [18–99]	62.3 [30–100]	1. standard dosage regimen of 70/50 mg; 2. Patients with hepatic insufficiency received 70/35 mg; 3. patients > 75 kg received 70/70 mg	LC-MS [NR]

allo-HSCT: allogeneic hematopoietic stem cell transplantation; ECMO: Extracorporeal membrane oxygenation; HTx: Heart transplantation; IFIs: invasive fungal infections; LT: Liver transplantation; PF: peritoneal fluid; NR: not reported.

**Table 2:** Modeling strategies and final pharmacokinetics parameters of included studies.

Study (Publication Year)	Software/ Algorithm	Fixed Effect Parameters (RSE)		Between-Subject Variability (%)	Residual Unexplained Variability	Internal Validation	External Validation	Simulation Target	Modeling Application
Würthwein et al. (2011) [32]	NONMEM/FOCE-I	CL (L/h)	=0.462	25	prop.err = 21%	VPC GOF Bootstrap	NR	NR	Evaluate covariate effects
		V_1_ (L)	=8.33	29					
		Q (L/h)	=1.25	/					
		V_2_ (L)	=3.59	/					
Würthwein et al. (2013) [28]	NONMEM/FOCE-I	CL (L/h)	=0.411 × [1 + 0.0102 × (BW-76)]	28.5	prop.err = 14.3%	pcVPC VPC GOF Bootstrap	36/456	NR	Evaluate covariate effects
		V_1_ (L)	=5.85 × [1 + 0.0102 × (BW-76)]	28.8					
		Q (L/h)	=0.843	/					
		V_2_ (L)	=6.53	66.8					
		IOV(CL)	=0.16	/					
		Cor-CL-V_1_	=0.802	/					
Pérez-Pitarch et al. (2018) [12]	NONMEM/FOCE-I	K_e_ (h^−1^)	=0.0899	11.8	add.error = 0.0941 mg/L	GOF Bootstrap VPC	NR	AUC/MIC: *C. albicans* 865; *C. glabrata* 450; *C. parapsilosis* 1185; C_max_/MEC: *Aspergillus* spp. 10–20	Evaluate the efficacy of different dosages
		V_1_ (L)	=6.46 × (BW/75) × [1–0.233 × (TP-5.6)]	21.4					
		K_12_ (h^−1^)	=0.494	/					
		K_21_ (h^−1^)	=0.392	/					
Yang et al. (2019) [27]	NONMEM/FOCE-I	CL (L/h)	=0.165 × (BSA/0.79)^1.3^	24.2	prop.err = 19.6%	GOF VPC NPDEs Bootstrap	NR	C_min_	Evaluate the efficacy of the dosing regimen; describe PK in a specific population
		V_1_ (L)	=1.730 × (BSA/0.79)^1.5^	/					
		Q (L/h)	=0.351	161.6					
		V_2_ (L)	=0.943	76.6					
Wang et al. (2020) [30]	NONMEM/NR	CL (L/h)	=0.21 × (OPT/5)^1.3^	20	Add.error = 0.73 mg/L	GOF VPC Bootstrap	NR	NR	Evaluate covariate effects; describe PK in a specific population
		V_1_ (L)	=(2.21 + SEX × 0.62) × (OPT/5)^0.93^	10					
		V_2_ (L)	=2.87	48.0					
		Q (L/h)	=0.84 × (SOFA/7)^1.98^	/					
Bailly et al. (2020) [31]	Monolix/SEAM	CL (L/h)	=0.98	42.3	prop.err = 12.2%	GOF VPC	NR	AUC/MIC: 50, 450, 865; C_max_/MIC 5, 10, 15, 20	Evaluate different dosages
		V_1_ (L)	=9.01	42.6					
		Q (L/h)	=5.12	79.9					
		V_2_ (L)	=11.9	77.2					
Niu et al. (2020) [25]	Phoenix NLIME	CL (L/h)	=0.1 × (BSA/0.79)^0.89^ × (lnAST/3.38)^−0.23^	33.3	prop.err = 26.6%	GOF VPC NPDEs Bootstrap	NR	AUC_24_/MIC	Dosing optimization against Candida spp.
		V_1_ (L)	=1.36 × (BSA/0.79)	32.9					
Borsuk-De Moor et al. (2021) [11]	NONMEM/FOCE-I	CL (L/h)D1	=0.563 × (BW/70)^0.75^	24.7	prop.err = 19.9%	pcVPC GOF Bootstrap	NR	AUC_24_/MIC *C.albicans* 865; *C. glabrata* 450; *C.parapsilosis* 1185	Evaluate covariate effects; describe PK in a specific population
		CL (L/h)D2	=0.737 * (BW/70)^0.75^	24.7					
		CL (L/h)D3	=1.01 × (BW/70)^0.75^	24.7					
		V_1_ (L) D1	=6.04 × (BW/70)	28.6					
		V_1_ (L) D2	=7.32 × (BW/70)	28.6					
		V_1_ (L) D3	=7.70 × (BW/70)	28.6					
		Q (L/h)	=1.31	/					
		V_2_ (L)	=5.13	49.4					
		Cor-CL-V1	=0.868	/					
Li et al. (2021) [23]	NONMEM/FOCE-I	CL (L/h)	= 0.323 × 0.89 × (35/ALB)^1.27^ (TBIL ≤ 22 μmol/L) = 0.323 × (35/ALB)^1.27^ × (TBIL/22)^0.265^ (TBIL >22 μmol/L)	22.4	prop.err = 24%	pcVPC GOF NPDEs	NR	AUC_24_/MIC *C. albicans* 865; *C. glabrata* 450; *C. parapsilosis* 1185	Evaluate covariate effects
		V_1_ (L)	=6.77 × (WT/70)^1.08^	/					
		Q (L/h)	=0.923	/					
		V_2_ (L)	=4.58	/					
Gastine et al. (2022) [33]	NONMEM/FOCE-I	CL (L/h/70 kg)	=0.790	27.5	prop.err = 19.4%	GOF VPC Bootstrap	NR	AUC_24_/MIC: *C. albicans* 865; *C. glabrata* 450; *C. parapsilosis* 1185; fAUC_24_/MIC: (10–20)	Assess extended twice-weekly dosage using caspofungin
		V_1_ (L/70 kg)	=7.75	31.5					
		Q (L/h/70 kg)	=1.20	/					
		V_2_ (L/70 kg)	=5.29	15.1					
		IOV(CL)	=17.2%	/					
Wu et al. (2022) [29]	NONMEM/NR	CL (L/h)	=0.385 × (ALB/37.42)^−1.01^	33.5	prop.err = 13.4% add.error = 0.213 mg/L	GOF Bootstrap VPC NPDE	NR	AUC_24_/MIC: *C. albicans* 865; *C. glabrata* 450; *C. parapsilosis* 1185	Evaluate covariate effects
		V_1_ (L)	=4.27	67.5					
		Q (L/h)	=2.85	0.0					
		V_2_ (L)	=6.01	47.7					
Pressiat et al. (2022) [26]	Monolix/ FOCE-I	CL (L/h)	=0.38	33.0	prop.err = 36%	GOF pcVPC	NR	fAUC_24_/MIC: *C. albicans* 25.9; *C. glabrata* 13.5; *C. parapsilosis* 35.5	Analyze the PK/PD of caspofungin in a specific population
		V_1_ (L)	=6.24	59.0					
		Q (L/h)	=2.58	/					
		V_2_ (L)	=6.44	107.0					
		K_eff,13_	=0.08	54					
		K_eff,31_	=0.26	/					
Yang et al. (2022) [34]	NONMEM/FOCE-I	CL (L/h)	=0.32 × (1 + 0.46 × ALB*) × (1 + 0.98 × WT*) (ALB* = 1, ALB < 35 g/L; ALB* = 0, ALB ≥ 35 g/L; WT* = 1, WT ≥ 70 kg, WT* = 0, WT< 70 kg)	29.2	prop.err = 19.3%	Bootstrap GOF pcVPC	NR	fAUC_24_/MIC *C. albicans* 20; *C. glabrata* 7; *C. parapsilosis* 7	Evaluate covariate effects; describe PK in a specific population
		V_1_ (L)	=13.31 × (1 + 0.49 × ALB*) × (1 + 0.24 × WT*) (ALB* = 1, ALB < 35 g/L; ALB* = 0, ALB ≥ 35 g/L; WT* = 1, WT ≥ 70 kg, WT* = 0, WT< 70 kg)	59.2					

CL: clearance; Q: intercompartmental clearance; V_1_: central volume of distribution; V_2_: peripheral volume of distribution.

**Table 3:** Investigated and identified covariates in population pharmacokinetic models of included studies.

Study (Publication Year)	Tested Covariates			Covariate Selection Criteria		Significant Covariates			
	Demographic	Laboratory Tests	Co-Administration	Forward Inclusion	Backward Elimination	CL	V1	Q	V2
Würthweinet al. (2011) [32]	Sex, Age, Weight, BSA	TBIL, CLCR	Liposomal amphotericin B	*p* < 0.001	*p* < 0.001	NR	NR	NR	NR
Würthwein et al. (2013) [28]	Dose, Sex, Age, Weight	TBIL, CLCR	NR	*p* < 0.05	*p* < 0.01	Weight	Weight	NR	NR
Pérez-Pitarch et al. (2018) [12]	Age, Sex, Weight	TP, SCR, TBIL, CLCR	NR	*p* < 0.05	*p* < 0.01	NR	Weight, TP	NR	NR
Yang et al. (2019) [27]	Age, Weight, BSA	SCR, ALB	NR	NR	*p* < 0.05	BSA	BSA	NR	NR
Wang et al. (2020) [30]	Age, Sex, Weight, BMI, ECMO, OPT, 24 h fluid balance	SOFA, ALT, AST, ALB, TBIL, CLCR, PCT	NR	*p* < 0.05	*p* < 0.01	OPT	Sex	SOFA	NR
Bailly et al. (2020) [31]	Age, Weight, ECMO	ALB, PAL, TBIL, AST, ALT, SCR, SOFA	NR	NR	NR	NR	NR	NR	NR
Niu et al. (2020) [25]	BSA, Weight,	CR, eGFR, ALB, TBIL, DBIL, ALT, AST, γ-GT	NR	*p* < 0.05	*p* < 0.01	BSA, AST	BSA	NR	NR
Borsuk-De Moor et al. (2021) [11]	Age, Weight, Height, Sex, ECMO, CRRT, Survival, dose	SOFA, PCT, UF, ELWI, Cardiac Output, ALB, TP, Liver Failure	NR	NR	*p* < 0.01	NR	NR	NR	NR
Li et al. (2021) [23]	Sex, Age, Weight	ALT, AST, TBIL, ALB, CLCR	NR	*p* < 0.05	*p* < 0.01	ALB, TBIL	Weight	NR	NR
Gastine et al. (2022) [33]	Weight, Age, BSA, Sex, Race	ALB, CLCR	Acyclovir, Vancomycin, Dexamethasone	*p* < 0.05	*p* < 0.01	Weight	Weight	Weight	Weight
Wu. et al. (2022) [29]	Age, Sex, Weight, Height, BMI, ECMO, CRRT	ALB, CLCR, AST, ALT, DBIL, TBIL, PCT, PLT, SCR, TP	NR	*p* < 0.05	*p* < 0.001	ALB	NR	NR	NR
Pressiat et al. (2022) [26]	Age, Sex, Weight, BMI	ALB, TP, TBIL, SCR, CLCR, SOFA, WBC, CRP, MELD, PT	NR	*p* < 0.05	NR	NR	NR	NR	NR
Yang et al. (2022) [34]	Weight, CMT, SOP, SOT, ICU	ALB, MM, CYC, MET	NR	*p* < 0.05	*p* < 0.001	Weight, ALB	Weight, ALB	NR	NR

BMI: body mass index; BSA: body surface area (calculated according to the formula of Mosteller); ALB: albumin; eGFR: the estimated glomerular filtration rate; CLCR: creatinine clearance (determined according to the formula of Cockcroft and Gault); SCR: serum creatinine; PCT: procalcitonin; TP: total protein; TBIL: total bilirubin; DBIL: direct bilirubin; Hb: hemoglobin; AST: aspartate aminotransferase; ALT: alanine aminotransferase; PAL: phenylalanine ammonia-lyase; ECMO: extracorporeal membrane oxygenation; CRRT: continuous renal replacement therapy; SOFA: sequential organ failure assessment; OPT: operative time; ELWI, extravascular lung water index; UF: ultrafiltration rate; PLT: platelet; WBC: leukocyte count; CRP: C-reactive protein; MELD: model for end-stage liver disease; PT: prothrombin time; HT: heart transplantation; NR: not reported.

## Data Availability

The data generated during and/or analyzed during the current study are available from the published literature.

## Supplementary Materials

The following supporting information can be downloaded at: https://www.mdpi.com/article/10.3390/pharmaceutics16060819/s1, Figure S1: The steady-state concentration-time profiles of piperacillin for (a) infants, (b) children, and (c) adults when infused intermittently. The solid line represents median of the simulated concentration-time profile. The light shadows represent the 10th–90th percentiles of the simulated concentration-time profiles. All virtual patients were assumed to be male received caspofungin at a dose of 70 mg on the first day, followed by 50 mg once a day as infusion for 1 h for adults and 70 mg/m^2^ on the first day, followed by 50 mg/m^2^ once a day as infusion for 1h for infants and children. Figure S2: Check List.

## References

[1] Pappas P.G., Kauffman C.A., Andes D.R., Clancy C.J., Marr K.A., Ostrosky-Zeichner L., Reboli A.C., Schuster M.G., Vazquez J.A., Walsh T.J. (2016). Clinical Practice Guideline for the Management of Candidiasis: 2016 Update by the Infectious Diseases Society of America. Clin. Infect. Dis..

[2] Hope W.W., Castagnola E., Groll A.H., Roilides E., Akova M., Arendrup M.C., Arikan-Akdagli S., Bassetti M., Bille J., Cornely O.A. (2012). ESCMID* guideline for the diagnosis and management of Candida diseases 2012: Prevention and management of invasive infections in neonates and children caused by *Candida* spp. Clin. Microbiol. Infect..

[3] HIGHLIGHTS OF PRESCRIBING INFORMATION for CANCIDAS-Merck. https://www.accessdata.fda.gov/drugsatfda_docs/label/2021/021227Orig1s040lbl.pdf.

[4] Sinnollareddy M.G., Roberts J.A., Lipman J., Akova M., Bassetti M., De Waele J.J., Kaukonen K.M., Koulenti D., Martin C., Montravers P. (2015). Pharmacokinetic variability and exposures of fluconazole, anidulafungin, and caspofungin in intensive care unit patients: Data from multinational Defining Antibiotic Levels in Intensive care unit (DALI) patients Study. Crit. Care.

[5] van der Elst K.C., Veringa A., Zijlstra J.G., Beishuizen A., Klont R., Brummelhuis-Visser P., Uges D.R., Touw D.J., Kosterink J.G., van der Werf T.S. (2017). Low Caspofungin Exposure in Patients in Intensive Care Units. Antimicrob. Agents Chemother..

[6] van Vianen W., de Marie S., ten Kate M.T., Mathot R.A., Bakker-Woudenberg I.A. (2006). Caspofungin: Antifungal activity in vitro, pharmacokinetics, and effects on fungal load and animal survival in neutropenic rats with invasive pulmonary aspergillosis. J. Antimicrob. Chemother..

[7] Ambrose P.G., Bhavnani S.M., Rubino C.M., Louie A., Gumbo T., Forrest A., Drusano G.L. (2007). Pharmacokinetics-pharmacodynamics of antimicrobial therapy: It’s not just for mice anymore. Clin. Infect. Dis..

[8] Wiederhold N.P., Kontoyiannis D.P., Chi J., Prince R.A., Tam V.H., Lewis R.E. (2004). Pharmacodynamics of caspofungin in a murine model of invasive pulmonary aspergillosis: Evidence of concentration-dependent activity. J. Infect. Dis..

[9] Louie A., Deziel M., Liu W., Drusano M.F., Gumbo T., Drusano G.L. (2005). Pharmacodynamics of caspofungin in a murine model of systemic candidiasis: Importance of persistence of caspofungin in tissues to understanding drug activity. Antimicrob. Agents Chemother..

[10] Andes D., Diekema D.J., Pfaller M.A., Bohrmuller J., Marchillo K., Lepak A. (2010). In vivo comparison of the pharmacodynamic targets for echinocandin drugs against Candida species. Antimicrob. Agents Chemother..

[11] Borsuk-De Moor A., Sysiak-Sławecka J., Rypulak E., Borys M., Piwowarczyk P., Raszewski G., Onichimowski D., Czuczwar M., Wiczling P. (2020). Nonstationary Pharmacokinetics of Caspofungin in ICU Patients. Antimicrob. Agents Chemother..

[12] Pérez-Pitarch A., Ferriols-Lisart R., Aguilar G., Ezquer-Garín C., Belda F.J., Guglieri-López B. (2018). Dosing of caspofungin based on a pharmacokinetic/pharmacodynamic index for the treatment of invasive fungal infections in critically ill patients on continuous venovenous haemodiafiltration. Int. J. Antimicrob. Agents.

[13] Sheiner L.B., Beal S., Rosenberg B., Marathe V.V. (1979). Forecasting individual pharmacokinetics. Clin. Pharmacol. Ther..

[14] Page M.J., McKenzie J.E., Bossuyt P.M., Boutron I., Hoffmann T.C., Mulrow C.D., Shamseer L., Tetzlaff J.M., Akl E.A., Brennan S.E. (2021). The PRISMA 2020 statement: An updated guideline for reporting systematic reviews. BMJ.

[15] Liu X., Ju G., Yang W., Chen L., Xu N., He Q., Zhu X., Ouyang D. (2023). Escitalopram Personalized Dosing: A Population Pharmacokinetics Repository Method. Drug Des. Devel. Ther..

[16] Jamsen K.M., McLeay S.C., Barras M.A., Green B. (2014). Reporting a population pharmacokinetic-pharmacodynamic study: A journal’s perspective. Clin. Pharmacokinet..

[17] Kanji S., Hayes M., Ling A., Shamseer L., Chant C., Edwards D.J., Edwards S., Ensom M.H., Foster D.R., Hardy B. (2015). Reporting Guidelines for Clinical Pharmacokinetic Studies: The ClinPK Statement. Clin. Pharmacokinet..

[18] Fancher K.M., Sacco A.J., Gwin R.C., Gormley L.K., Mitchell C.B. (2016). Comparison of two different formulas for body surface area in adults at extremes of height and weight. J. Oncol. Pharm. Pract..

[19] Li Z.R., Wang C.Y., Zhu X., Jiao Z. (2021). Population Pharmacokinetics of Levetiracetam: A Systematic Review. Clin. Pharmacokinet..

[20] Lewis R.E. (2011). Current concepts in antifungal pharmacology. Mayo Clin. Proc..

[21] Ernst E.J., Klepser M.E., Ernst M.E., Messer S.A., Pfaller M.A. (1999). In vitro pharmacodynamic properties of MK-0991 determined by time-kill methods. Diagn. Microbiol. Infect. Dis..

[22] CLSI (2022). Performance Standards for Antifungal Susceptibility Testing of Yeasts.

[23] Li F., Zhou M., Jiao Z., Zou Z., Yu E., He Z. (2021). Caspofungin pharmacokinetics and probability of target attainment in ICU patients in China. J. Glob. Antimicrob. Resist..

[24] Mori M., Imaizumi M., Ishiwada N., Kaneko T., Goto H., Kato K., Hara J., Kosaka Y., Koike K., Kawamoto H. (2015). Pharmacokinetics, efficacy, and safety of caspofungin in Japanese pediatric patients with invasive candidiasis and invasive aspergillosis. J. Infect. Chemother..

[25] Niu C.H., Xu H., Gao L.L., Nie Y.M., Xing L.P., Yu L.P., Wu S.L., Wang Y. (2020). Population Pharmacokinetics of Caspofungin and Dosing Optimization in Children with Allogeneic Hematopoietic Stem Cell Transplantation. Front. Pharmacol..

[26] Pressiat C., Ait-Ammar N., Daniel M., Hulin A., Botterel F., Levesque E. (2022). Pharmacokinetics/Pharmacodynamics of Caspofungin in Plasma and Peritoneal Fluid of Liver Transplant Recipients. Antimicrob. Agents Chemother..

[27] Yang X.M., Leroux S., Storme T., Zhang D.L., de Beaumais T.A., Shi H.Y., Yang Y.L., Wang X.L., Zhao W., Jacqz-Aigrain E. (2019). Body Surface Area-Based Dosing Regimen of Caspofungin in Children: A Population Pharmacokinetics Confirmatory Study. Antimicrob. Agents Chemother..

[28] Würthwein G., Cornely O.A., Trame M.N., Vehreschild J.J., Vehreschild M.J., Farowski F., Müller C., Boos J., Hempel G., Hallek M. (2013). Population pharmacokinetics of escalating doses of caspofungin in a phase II study of patients with invasive aspergillosis. Antimicrob. Agents Chemother..

[29] Wu Z., Lan J., Wang X., Wu Y., Yao F., Wang Y., Zhao B.X., Wang Y., Chen J., Chen C. (2022). Population Pharmacokinetics of Caspofungin and Dose Simulations in Heart Transplant Recipients. Antimicrob. Agents Chemother..

[30] Wang Q., Zhang Z., Liu D., Chen W., Cui G., Li P., Zhang X., Li M., Zhan Q., Wang C. (2020). Population Pharmacokinetics of Caspofungin among Extracorporeal Membrane Oxygenation Patients during the Postoperative Period of Lung Transplantation. Antimicrob. Agents Chemother..

[31] Bailly S., Gautier-Veyret E., Lê M.P., Bouadma L., Andremont O., Neuville M., Mourvillier B., Sonneville R., Magalhaes E., Lebut J. (2020). Impact of Loading Dose of Caspofungin in Pharmacokinetic-Pharmacodynamic Target Attainment for Severe Candidiasis Infections in Patients in Intensive Care Units: The CASPOLOAD Study. Antimicrob. Agents Chemother..

[32] Würthwein G., Young C., Lanvers-Kaminsky C., Hempel G., Trame M.N., Schwerdtfeger R., Ostermann H., Heinz W.J., Cornely O.A., Kolve H. (2012). Population pharmacokinetics of liposomal amphotericin B and caspofungin in allogeneic hematopoietic stem cell recipients. Antimicrob. Agents Chemother..

[33] Gastine S., Hempel G., Neely M.N., Walsh T.J., Groll A.H. (2022). Pharmacokinetic modelling of caspofungin to develop an extended dosing regimen in paediatric patients. J. Antimicrob. Chemother..

[34] Yang Q., Zhang T., Zhang Y., Sun D., Zheng X., Du Q., Wang X., Cheng X., Xing J., Dong Y. (2022). The recommended dosage regimen for caspofungin in patients with higher body weight or hypoalbuminaemia will result in low exposure: Five years of data based on a population pharmacokinetic model and Monte-Carlo simulations. Front. Pharmacol..

[35] Sandhu P., Lee W., Xu X., Leake B.F., Yamazaki M., Stone J.A., Lin J.H., Pearson P.G., Kim R.B. (2005). Hepatic uptake of the novel antifungal agent caspofungin. Drug Metab. Dispos..

[36] Stader F., Wuerthwein G., Groll A.H., Vehreschild J.J., Cornely O.A., Hempel G. (2015). Physiology-based pharmacokinetics of caspofungin for adults and paediatrics. Pharm. Res..

[37] Walsh T.J., Adamson P.C., Seibel N.L., Flynn P.M., Neely M.N., Schwartz C., Shad A., Kaplan S.L., Roden M.M., Stone J.A. (2005). Pharmacokinetics, safety, and tolerability of caspofungin in children and adolescents. Antimicrob. Agents Chemother..

[38] Sáez-Llorens X., Macias M., Maiya P., Pineros J., Jafri H.S., Chatterjee A., Ruiz G., Raghavan J., Bradshaw S.K., Kartsonis N.A. (2009). Pharmacokinetics and safety of caspofungin in neonates and infants less than 3 months of age. Antimicrob. Agents Chemother..

[39] Sinha J., Al-Sallami H.S., Duffull S.B. (2019). Choosing the Allometric Exponent in Covariate Model Building. Clin. Pharmacokinet..

[40] Fortmann I., Hartz A., Paul P., Pulzer F., Müller A., Böttger R., Proquitté H., Dawczynski K., Simon A., Rupp J. (2018). Antifungal Treatment and Outcome in Very Low Birth Weight Infants: A Population-based Observational Study of the German Neonatal Network. Pediatr. Infect. Dis. J..

[41] Roch A., Woloch C., Blayac D., Solas C., Quaranta S., Mardelle V., Castanier M., Papazian L., Sampol-Manos E. (2011). Effect of fluid loading during hypovolaemic shock on caspofungin pharmacokinetic parameters in pig. Crit. Care.

[42] Roberts J.A., Abdul-Aziz M.H., Lipman J., Mouton J.W., Vinks A.A., Felton T.W., Hope W.W., Farkas A., Neely M.N., Schentag J.J. (2014). Individualised antibiotic dosing for patients who are critically ill: Challenges and potential solutions. Lancet Infect. Dis..

[43] Gasperetti T., Welte R., Oberacher H., Marx J., Lorenz I., Schellongowski P., Staudinger T., Burgmann K., Eller P., Santner T. (2021). Penetration of echinocandins into wound secretion of critically ill patients. Infection.

[44] Betts R.F., Nucci M., Talwar D., Gareca M., Queiroz-Telles F., Bedimo R.J., Herbrecht R., Ruiz-Palacios G., Young J.A., Baddley J.W. (2009). A Multicenter, double-blind trial of a high-dose caspofungin treatment regimen versus a standard caspofungin treatment regimen for adult patients with invasive candidiasis. Clin. Infect. Dis..

[45] Kurland S., Furebring M., Löwdin E., Eliasson E., Nielsen E.I., Sjölin J. (2019). Pharmacokinetics of Caspofungin in Critically Ill Patients in Relation to Liver Dysfunction: Differential Impact of Plasma Albumin and Bilirubin Levels. Antimicrob. Agents Chemother..

[46] Liu X., Ju G., Huang X., Yang W., Chen L., Li C., He Q., Xu N., Zhu X., Ouyang D. (2024). Escitalopram population pharmacokinetics and remedial strategies based on CYP2C19 phenotype. J. Affect. Disord..

[47] Kurland S., Löwdin E., Furebring M., Shams A., Chryssanthou E., Lagerbäck P., Tängden T., Breuer O., Sjölin J. (2022). Human plasma protein levels alter the in vitro antifungal activity of caspofungin: An explanation to the effect in critically ill?. Mycoses.

