# Supplementary material for: Development and Quality Control of a Population Pharmacokinetic Model Library for Caspofungin
Source: Pharmaceutics. 2024 Jun 17;16(6):819. doi: 10.3390/pharmaceutics16060819 (PMC11207296; doi:10.3390/pharmaceutics16060819)
Supplement: Supplementary file 1 [file pharmaceutics-16-00819-s001.zip › pharmaceutics-3021266-supplementary.pdf]

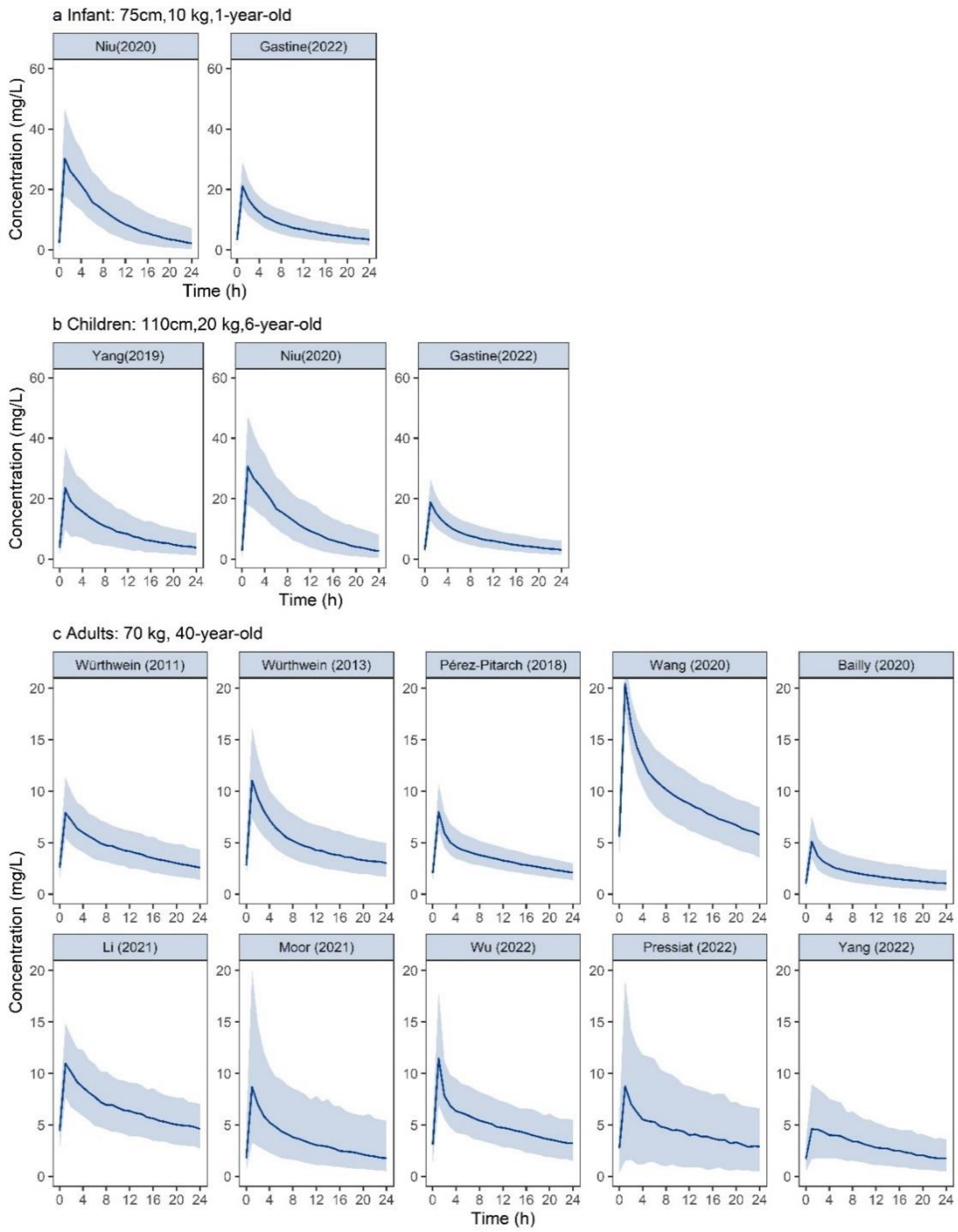

Figure S1: The steady-state concentration-time profiles of piperacillin for (a) infants, (b) children, and (c) adults when infused intermittently. The solid line represents median of the simulated concentration-time profile. The light shadows represent the 10th-90th percentiles of the simulated concentration-time profiles. All virtual patients were assumed to be male received caspofungin at a dose of 70mg on the first day, followed by 50 mg once a day as infusion for 1h for adults and $70 mg/m^2$ on the first day, followed by $50 mg/m^2$ once a day as infusion for 1h for infants and children.

Section	Suggested contents
Introduction	Motivation for and objective(s) of the pharmacometrics research
Methods	Sufficient information for the general reader to repeat the study:<br>Ethics approval, Study population,<br>Dosing, Sampling schedule<br>Analytical methods and lower limit of quantification<br>Pharmacokinetic or pharmacokinetic<br>pharmacodynamic modelling strategy:<br>– Candidate structural models<br>– Distribution of individual model parameters<br>– Residual error structure<br>– Methods for handling missing data<br>– Methods for base model determination<br>– Methods for base model evaluation<br>– Covariate analysis strategy<br>– Methods for final model evaluation<br>– Software package(s) used for the analysis<br>– Estimation method(s) used
Results	Numbers of individuals and observations included in the analysis<br>Table of patient demographic and clinical variables<br>Plot of concentrations versus time and/or effects versus concentrations<br>Summary of the model-building process and the derived final model Schematic of the final model, Table of the final model parameters, Final model evaluation plots
Discussion	Highlight what the study achieved, implications of the work suggestions for future work and limitations of the study
Conclusion	Briefly answer questions posed by the objectives

Figure S2： Check List

The search terms utilized for compiling the model library:

Pubmed: (“Caspofungun" or “cancidas" or “MK 0991" or “L 743,872") AND ("population pharmacokinetic" or "nonlinear mixed effect model" or "NONMEM" or "Pmetrics" or "WINNONMIX" or "ADAPT" or "P-PHARM" or "nlmixr" or "NLME" or "USC*PACK" or "MONOLIX")

Scopus: (TITLE-ABS-KEY (caspofungin) OR TITLE-ABS-KEY (cancidas) OR TITLE-ABS-KEY (mk 0991) OR TITLE-ABS-KEY (l 743872)) AND (TITLE-ABS-KEY (population AND pharmacokinetics) OR TITLE-ABS-KEY (nonlinear AND mixed AND effect AND model) OR TITLE-ABS-KEY (nonmem) OR TITLE-ABS-KEY (pmetrics) OR TITLE-ABS-KEY (winnonmix) OR TITLE-ABS-KEY (adapt) OR TITLE-ABS-KEY (p-pharm) OR TITLE-ABS-KEY (nlmixr) OR TITLE-ABS-KEY (nlme) OR TITLE-ABS-KEY (monolix))

Web of Science: (TS=(Caspofungin) OR TS=(cancidas) OR TS=(MK 0991) OR TS=(L743,872)) AND (TS=(population pharmacokinetic) OR TS=(nonlinear mixed effect model)OR TS=(NONMEM) OR TS=(Pmetrics) OR TS=(WINNONMIX) OR TS=(ADAPT) OR TS=(P-PHARM) OR TS=(nlmixr) OR TS=(NLME) OR TS=(MONOLIX))

Embase: (Caspofungin or cancidas or “MK 0991” or L43,872) and (population pharmacokinetics or nonlinear mixed effect model or NONMEM or Pmetrics or WINNONMEX or ADAPT or P-PHARM or nlmixr or USC*PACK or MONOLIX)

Code:

```
#AiM: Establish a model repository of parametric PPK models for Caspofungin using rxode2
#Author:Nuo Xu
#Update data:2022/11/08
#Connect:nuoxu22@m.edu.cn

rm(list=ls())
#set working directory to current folder
curr.dir<-dirname(rstudioapi::getActiveDocumentContext()$path)
setwd(curr.dir)
# create the result folder
output_dir <- "Result"
if (!file.exists(output_dir)) {dir.create(output_dir)}

library(tidyverse) # for data visualization and manipulation
library(rxode2) # for simulation
library(patchwork)
library(cowplot)

#----------------------------------------
#----------Adults 70KG    40Y    ------------------------
#----------------------------------------
# Define the 2-comp model,model info
# CL（L/h）=0.462
# V1（L）     =8.33
# Q    (L/h) =1.25
# V2 (L)      =3.59
# IIV(CL)    =25%
# IIV(V1)    =29%
# Proportional error=0.21
# BW(kg)

# Typical population:
# Adults Allogeneic Hematopoietic Stem Cell Recipients
# age : 40 years old
# BW    : 70kg
# Dose: 70 mg on day 1,followed by 50 mg QD,Infusion for 1h

set.seed(123456)

# 2-comp model
mod1 <- RxODE({
   CL     = TVCL*exp(eta.CL);
   V1     = TVV1*exp(eta.V1);
```

```
  Q     = TVQ;
  V2    = TVV2;

  C1    = centr/V1;
  C2    = peri/V2;

  d/dt(centr) = - CL*C1 - Q*C1 + Q*C2;
  d/dt(peri)  = Q*C1 - Q*C2;

  cp = C1*(1 + prop.err.sd)
})

# Adults
# Define typical patient: adults
BW   <- 70     # kg
AGE <- 40     # years old

# Define fixed effect parameters
theta1<- c(TVCL=0.462, TVV1=8.33, TVQ=1.25, TVV2=3.59)

# Define between subject variability
omega1 <- lotri(eta.CL ~ 0.25^2, eta.V1 ~ 0.29^2)

# Define unexplained variability
sigma1 <- lotri(prop.err.sd ~ 0.21^2)

# DOSE of CAS(iv)
dose_cas_day1 <-70     # mg 1-h infusion
dose_cas_later<-50     # mg 1-h infusion

# Define event record
ev1 <- et(amountUnits="mg", timeUnits="hours") %>%
  add.dosing(dosing.to = "centr",dose = dose_cas_day1, rate = dose_cas_day1/1,
             nbr.doses = 1, start.time = 0) %>%
  add.dosing(dosing.to = "centr",dose = dose_cas_later, rate = dose_cas_later/1,
             nbr.doses = 3, dosing.interval = 24,start.time = 24) %>%
# sampling:0-24h after the last dosing
  add.sampling(seq(from=72,to=96,by=1))

# Perform simulation
# total number of subject: 1000
sim1   <- rxSolve(mod1,theta1,ev1,omega=omega1,sigma=sigma1,nSub=1000)
```

```
# Concentration-Time plot
pk_CAS1 <- sim1 %>%
   group_by(time) %>%
   # 10-90% Prediction interval
   summarise(medCL = median(CL),
                  medconc = median(cp),
                  lowconc = quantile(cp,0.1),
                  highconc = quantile(cp, 0.9)) %>%
   ungroup() %>%
   # time after last dose
   mutate(time = as.numeric(time),
              tald = time-72)

dat1 <- pk_CAS1 %>%
   mutate(study="Würthwein et al.(2011)",
              drug="CAS",
              pop="Adult")


# 2----Würthwein et al.(2013)----
# Define the 2-comp model,model info

# CL = 0.411*(1+0.0102*(BW-76)) (L/h)
# V1 = 5.85*(1+0.0102*(BW-76)) (L)
# Q    = 0.843 (L/h)
# V2 = 6.53 (L)

# IIV(CV%): CL = 0.285, V1 = 0.288, V2 = 0.668
# 0.285=sqrt(exp(omega^2)-1)

# omega^2=log(0.285^2+1)=0.0781
# IOV(CV%): CL = 0.16
# prop.err = 0.143
# Correlation for CL-V1: 0.802

# omega1,2=omega2,1/(sqrt(omega1,1^2)*sqrt(omega2,2^2))
# omega2,1=0.802*sqrt(0.0781)*sqrt(0.079) =0.06299607
# omega2,1^2=0.003968505=0.00397

# Typical population:
# Adults Allogeneic Hematopoietic Stem Cell Recipients
# BW :    70kg
# age : 40 years old
# Dose: 70 mg on day 1,followed by 50 mg QD,Infusion for 1h
```

```
set.seed(1234567)

pop_par <- c(0.411,      # (L/h), CL
             5.85,       # (L), V1
             0.843,      # (L/h), Q
             6.53)       # (L), V2

eta2.cl <- 0.0781 # omega^2=log(0.285^2+1)
eta2.v1 <- 0.0797 # omega^2=log(0.288^2+1)
eta2.v2 <- 0.3690 # omega^2=log(0.668^2+1)
iov2.cl <- 0.0253 # omega^2=log(0.16^2+1)
cov2.cl_v1 <- 0.06327 # 0.802=omega2,1/(sqrt(omega1,1^2)*sqrt(omega2,2^2))
prop.err <- 0.143

# dose regimen
# an initial dose of 70 mg followed by
# a maintenance dose of 50 mg/24 h infused
# over a 1-hour period
intialdose <- 70 # mg
maintaindose <- 50 # mg
dur <- 1 # 1 h
dose_days <- 4 # 4 days
interval <- 24 # 24 h
nsub <- 1000
WT <- 70

# 1. Create virtual patients
covar <- data.frame(id = c(1:nsub),
                    WT = WT)

# 2. Define PPK model of Caspofungin
# a 2-comp model with the first order elimination
mod2 <- RxODE({
   CL     = TVCL*exp(eta.CL+iov.CL1*occ1+iov.CL2*occ2+iov.CL3*occ3+iov.CL4*occ4);
   V1     = TVV1*exp(eta.V1);
   Q      = TVQ;
   V2     = TVV2*exp(eta.V2);

   C1 = centr/V1;
   C2 = peri/V2;
   d/dt(centr) = - CL*C1 - Q*C1 + Q*C2;
   d/dt(peri)   = Q*C1 - Q*C2;
```

```
  cp = C1*(1 + prop.err.sd);
})


# Define fixed effect parameters
theta2 <- c(TVCL=pop_par[1]*(1+0.0102*(WT-76)),
            TVV1=pop_par[2]*(1+0.0102*(WT-76)),
            TVQ=pop_par[3],
            TVV2=pop_par[4])

# Define between subject variability
omega2 <- lotri(lotri(eta.CL + eta.V1 + eta.V2 ~
                      c(eta2.cl,                              # IIV CL
                        cov2.cl_v1,   eta2.v1,                # IIV V1
                        0,            0,        eta2.v2)) ,   # IIV V2
                lotri(iov.CL1 ~ iov2.cl),                     # IOV CL
                lotri(iov.CL2 ~ iov2.cl),
                lotri(iov.CL3 ~ iov2.cl),
                lotri(iov.CL4 ~ iov2.cl))

# Define unexplained variability
sigma2 <- lotri(prop.err.sd ~ prop.err^2)

# DOSE of CAS(iv)
dose_cas_day1 <-70     # mg 1-h infusion
dose_cas_later<-50     # mg 1-h infusion

# define event record
ev2 <- et(amount.units = "mg", time.units = "hours") %>%
  add.dosing(dosing.to = "centr",dose = dose_cas_day1, rate = dose_cas_day1/1,
             nbr.doses = 1, start.time = 0) %>%
  add.dosing(dosing.to = "centr",dose = dose_cas_later, rate = dose_cas_later/1,
             nbr.doses = 3, dosing.interval = 24,start.time = 24) %>%
  # sampling:0-24h after the last dosing
  add.sampling(seq(from=72,to=96,by=1)) %>%
  mutate(occ=cumsum(!is.na(amt))) %>%
  mutate(occ1=case_when(occ==1 ~ 1,
                        TRUE ~ 0),
         occ2=case_when(occ==2 ~ 1,
                        TRUE ~ 0),
         occ3=case_when(occ==3 ~ 1,
                        TRUE ~ 0),
         occ4=case_when(occ==4 ~ 1,
                        TRUE ~ 0)) %>%
```

```
  as.data.frame() %>%
  as_tibble
sim2 <- rxSolve(mod2, theta2, ev2, omega=omega2, sigma=sigma2,nSub=1000)

# Concentration-Time plot
pk_CAS2 <- sim2 %>%
  group_by(time) %>%
  # 10-90% Prediction interval
  summarise(medCL = median(CL),
            medconc = median(cp),
            lowconc = quantile(cp,0.1),
            highconc = quantile(cp, 0.9)) %>%
  ungroup() %>%
  # time after last dose
  mutate(time = as.numeric(time),
         tald = time-72)

dat2 <- pk_CAS2 %>%
  mutate(study="Würthwein et al.(2013)",
         drug="CAS",
         pop="Adult")

# 3---- Pérez-Pitarch et al. (2018)----
# Define the 2-comp model,model info

# kel（h-1）=0.0899（6%）
# Vc（L）      =6.46（7%）
# k12（h-1）=0.494（13%）
# k12（h-1）=0.392（12%）

# ETA(kel) = 0.0140
# ETA(Vc)   = 0.0460

# Additive error=0.0941（6%）
# WT(kg)

# Typical population:
# Adults
# AGE : 40 years old
# BW   : 70 kg
# Dose: 70 mg on day 1,followed by 50 mg QD,Infusion for 1h

set.seed(12345678)
```

```
# 2-comp model
mod3 <- RxODE({
  ke     = TVke*exp(eta.ke);
  V1     = TVV1*exp(eta.V1);
  k12    = TVk12;
  k21    = TVk21;

  CL     = V1*ke;
  V2     = V1*k12/k21;
  Q      = V1*k12;

  C1     = centr/V1;
  C2     = peri/V2;

  d/dt(centr) = - CL*C1 - Q*C1 + Q*C2;
  d/dt(peri)   = Q*C1 - Q*C2;

  cp = C1+add.err
})

# Adults
# Define typical patient: adults
BW   <- 70     # kg
AGE <- 40     # years old

# Define fixed effect parameters
theta3<- c(   TVke   =0.0899,
              TVV1   =6.46,
              TVk12 =0.494,
              TVk21 =0.392)

# Define between subject variability
omega3 <- lotri(eta.ke ~ 0.0140, eta.V1 ~ 0.0460)

# Define unexplained variability
sigma3 <- lotri(add.err ~ 0.0941)

# DOSE of CAS(iv)
dose_cas_day1 <-70     # mg 1-h infusion
dose_cas_later<-50     # mg 1-h infusion

# Define event record
ev3 <- et(amountUnits="mg", timeUnits="hours") %>%
  add.dosing(dosing.to = "centr",dose = dose_cas_day1, rate = dose_cas_day1/1,
```

```
                    nbr.doses = 1, start.time = 0) %>%
    add.dosing(dosing.to = "centr",dose = dose_cas_later, rate = dose_cas_later/1,
                    nbr.doses = 3, dosing.interval = 24,start.time = 24) %>%
    # sampling:0-24h after the last dosing
    add.sampling(seq(from=72,to=96,by=1))

# Perform simulation
# total number of subject: 1000
sim3    <- rxSolve(mod3,theta3,ev3,omega=omega3,sigma=sigma3,nSub=1000)

# Concentration-Time plot
pk_CAS3 <- sim3 %>%
    group_by(time) %>%
    # 10-90% Prediction interval
    summarise(medCL = median(CL),
                  medconc = median(cp),
                  lowconc = quantile(cp,0.1),
                  highconc = quantile(cp, 0.9)) %>%
    ungroup() %>%
    # time after last dose
    mutate(time = as.numeric(time),
              tald = time-72)

dat3 <- pk_CAS3 %>%
    mutate(study="Pérez-Pitarch et al. (2018)",
              drug="CAS",
              pop="Adult")

# 4----Wang et al.(2020)-----
# Define the 2-comp model,model info

# CL(L/h) =0.21*(OPT/5)^1.3(8%)
# Vc(L)      =(2.21+SEX*0.62)*(OPT/5)^0.93(5%)
# Vp(L)      =2.87(16%)
# Q(L/h)    =0.84*(SOFA/7)^1.98(11%)

# IIV(CL) =0.04(21.3%)
# IIV(Vc) =0.01(15.5%)
# IIV(Vp) =0.23(21.2%)

# add.error=0.73

# OPT : operative time(h)
# SEX : male,SEX=1;female,SEX=0
```

```
# SOFA: score for organ failure

# Typical population:
# Adults
# male SEX=1
# age : 40 years old
# OPT : 4.0h
# WT    : 70kg
# Dose: 70 mg on day 1,followed by 50 mg QD,Infusion for 1h

set.seed(123456789)

# 2-comp model
mod4 <- RxODE({
   CL      = TVCL*exp(iiv.CL);
   V1      = TVV1*exp(iiv.V1);
   Q       = TVQ;
   V2      = TVV2*exp(iiv.V2);

   C1      = centr/V1;
   C2      = peri/V2
   d/dt(centr) = - CL*C1 - Q*C1 + Q*C2;
   d/dt(peri)    = Q*C1 - Q*C2;

   cp = C1 + add.err.sd
})

# Adults
# Define typical patient: adults
OPT    <- 5      # h
SEX    <- 1      # male

# Define fixed effect parameters
theta4<- c(TVCL=0.21*(OPT/5)^1.3^(0.04),
                TVV1=(2.21+SEX*0.62)*(OPT/5)^0.93^(0.01),
                TVQ =0.84,
                TVV2=2.87)

# Define between subject variability
omega4 <- lotri(iiv.CL ~ 0.04, iiv.V1 ~ 0.01,iiv.V2 ~ 0.23)

# Define unexplained variability
sigma4 <- lotri(add.err.sd ~ 0.73)
```

```
# DOSE of CAS(iv)
dose_cas_day1 <-70      # mg 1-h infusion
dose_cas_later<-50      # mg 1-h infusion

# Define event record
ev4 <- et(amountUnits="mg", timeUnits="hours") %>%
  add.dosing(dosing.to = "centr",dose = dose_cas_day1, rate = dose_cas_day1/1,
             nbr.doses = 1, start.time = 0) %>%
  add.dosing(dosing.to = "centr",dose = dose_cas_later, rate = dose_cas_later/1,
             nbr.doses = 3, dosing.interval = 24,start.time = 24) %>%
  # sampling:0-24h after the last dosing
  add.sampling(seq(from=72,to=96,by=1))

# Perform simulation
# total number of subject: 1000
sim4    <- rxSolve(mod4,theta4,ev4,omega=omega4,sigma=sigma4,nSub=1000)

# Concentration-Time plot
pk_CAS4 <- sim4 %>%
  group_by(time) %>%
  # 10-90% Prediction interval
  summarise(medCL = median(CL),
            medconc = median(cp),
            lowconc = quantile(cp,0.1),
            highconc = quantile(cp, 0.9)) %>%
  ungroup() %>%
  # time after last dose
  mutate(time = as.numeric(time),
         tald = time-72)

dat4 <- pk_CAS4 %>%
  mutate(study="Wang et al.(2020)",
         drug="CAS",
         pop="Adult")

# 5----Bailly et al.(2020)----
# Define the 2-comp model,model info

# CL(L/h) =0.98(13%)
# V1(L)      =9.01(13.4%)
# V2(L)      =11.9(24%)
# Q(L/h)    =5.12(26.7%)

# IIV(CL) =0.423(23.1%)
```

```
# IIV(V1) =0.426(24.5%)
# IIV(V2) =0.772
# IIV(Q)    =0.799

# prop error=0.122

# Typical population:
# Adults
# age : 40 years old
# BW    : 70kg
# Dose: 70 mg on day 1,followed by 50 mg QD,Infusion for 1h

set.seed(123456789)

# 2-comp model
mod5 <- RxODE({
   CL      = TVCL*exp(iiv.CL);
   V1      = TVV1*exp(iiv.V1);
   Q       = TVQ*exp(iiv.Q);
   V2      = TVV2*exp(iiv.V2);

   C1      = centr/V1;
   C2      = peri/V2
   d/dt(centr) = - CL*C1 - Q*C1 + Q*C2;
   d/dt(peri)    = Q*C1 - Q*C2;

   cp = C1*(1 + prop.err.sd)
})

# Adults
# Define typical patient: ICU patients
# BW : 70kg
# Age: 40 years old

# Define fixed effect parameters
theta5<- c(TVCL =0.98,
                  TVV1 =9.01,
                  TVQ    =5.12,
                  TVV2 =11.9)

# Define between subject variability
omega5 <- lotri(iiv.CL ~ 0.423^2, iiv.V1 ~ 0.426^2,iiv.V2 ~ 0.772^2,iiv.Q ~ 0.799^2)

# Define unexplained variability
```

```
sigma5 <- lotri(prop.err.sd ~ 0.122^2)

# DOSE of CAS(iv)
dose_cas_day1 <-70     # mg 1-h infusion
dose_cas_later<-50     # mg 1-h infusion

# Define event record
ev5 <- et(amountUnits="mg", timeUnits="hours") %>%
  add.dosing(dosing.to = "centr",dose = dose_cas_day1, rate = dose_cas_day1/1,
             nbr.doses = 1, start.time = 0) %>%
  add.dosing(dosing.to = "centr",dose = dose_cas_later, rate = dose_cas_later/1,
             nbr.doses = 3, dosing.interval = 24,start.time = 24) %>%
  # sampling:0-24h after the last dosing
  add.sampling(seq(from=72,to=96,by=1))

# Perform simulation
# total number of subject: 1000
sim5   <- rxSolve(mod5,theta5,ev5,omega=omega5,sigma=sigma5,nSub=1000)

# Concentration-Time plot
pk_CAS5 <- sim5 %>%
  group_by(time) %>%
  # 10-90% Prediction interval
  summarise(medCL = median(CL),
            medconc = median(cp),
            lowconc = quantile(cp,0.1),
            highconc = quantile(cp, 0.9)) %>%
  ungroup() %>%
  # time after last dose
  mutate(time = as.numeric(time),
         tald = time-72)

dat5 <- pk_CAS5 %>%
  mutate(study="Bailly et al.(2020)",
         drug="CAS",
         pop="Adult")

# 6----Li et al.(2021)----
# Define the 2-comp model,model info
# CL（L/h）=0.323（11%）
# V1（L）   =6.77(10%)
# Q   (L/h) =0.923(32%)
# V2 (L)    =4.12(25%)
```

```
#Effect of weight on V1                    =1.08(24%)
#Effect of ALB on CL                         =1.27(30%)
#Effect of TBIL(≤22μmol/L) on CL     =0.89(12%)
#Effect of TBIL(>22μmol/L) on CL     =0.265(32%)

# CL (L/h) = 0.323×0.89× (35/ALB)^1.27 (if TBIL ≤ 22 μmol/L)
# CL (L/h) = 0.323× (35/ALB)^1.27×(TBIL/22)^0.265 (if TBIL > 22 μmol/L)
# Vc (L)     = 6.77× (WT/70)^1.08
# Q (L/h)   = 0.923
# Vp (L)     = 4.58

# IIV(CL)   =0.224（18%）

# Proportional error=0.24（21%）
# BW(kg)

# Typical population:
# Adults Allogeneic Hematopoietic Stem Cell Recipients
# age : 40 years old
# BW   : 70kg
# Dose: 70 mg on day 1,followed by 50 mg QD,Infusion for 1h

set.seed(123456)

# 2-comp model
mod6 <- RxODE({
   CL     = TVCL*exp(iiv.CL);
   V1     = TVV1;
   Q      = TVQ;
   V2     = TVV2;

   C1     = centr/V1;
   C2     = peri/V2;

   d/dt(centr) = - CL*C1 - Q*C1 + Q*C2;
   d/dt(peri)   = Q*C1 - Q*C2;

   cp = C1*(1 + prop.err.sd)
})

# Adults
# Define typical patient: adults
BW   <- 70     # kg
ALB <- 35
```

```
TBIL<- 22      #μmol/L

# Define fixed effect parameters
theta6<- c(TVCL =0.323*0.89* (35/ALB)^1.27 ,
              TVV1 =6.77*(BW/70)^1.08,
              TVQ    =0.923,
              TVV2 =4.58)

# Define between subject variability
omega6 <- lotri(iiv.CL ~ 0.224^2)

# Define unexplained variability
sigma6 <- lotri(prop.err.sd ~ 0.24^2)

# DOSE of CAS(iv)
dose_cas_day1 <-70      # mg 1-h infusion
dose_cas_later<-50      # mg 1-h infusion

# Define event record
ev6 <- et(amountUnits="mg", timeUnits="hours") %>%
   add.dosing(dosing.to = "centr",dose = dose_cas_day1, rate = dose_cas_day1/1,
                  nbr.doses = 1, start.time = 0) %>%
   add.dosing(dosing.to = "centr",dose = dose_cas_later, rate = dose_cas_later/1,
                  nbr.doses = 3, dosing.interval = 24,start.time = 24) %>%
   # sampling:0-24h after the last dosing
   add.sampling(seq(from=72,to=96,by=1))

# Perform simulation
# total number of subject: 1000
sim6    <- rxSolve(mod6,theta6,ev6,omega=omega6,sigma=sigma6,nSub=1000)

# Concentration-Time plot
pk_CAS6 <- sim6 %>%
   group_by(time) %>%
   # 10-90% Prediction interval
   summarise(medCL = median(CL),
                 medconc = median(cp),
                 lowconc = quantile(cp,0.1),
                 highconc = quantile(cp, 0.9)) %>%
   ungroup() %>%
   # time after last dose
   mutate(time = as.numeric(time),
             tald = time-72)
```

```
dat6 <- pk_CAS6 %>%
  mutate(study="Li et al.(2021)",
         drug="CAS",
         pop="Adult")

# 7----Borsuk-De Moor et al.(2021)-----
# Define the 2-comp model,model info

# TVCL(L/h) =0.563(6.7%)
# TVV1(L)      =6.04(7.0 %)
# TVV2(L)      =5.13(13.7%)
# TVQ(L/h)    =1.31(15.7%)

# CL = TVCL*(BW/70)^0.75
# V    = TVV*(BW/70)^1

# IIV(CL) =0.247(27.3%)
# IIV(V1) =0.286.0(39.6%)
# IIV(V2) =0.494(37.1%)
# IIV(Q)    =0 FIX

# prop error = 0.199

# Typical population:
# Adults
# age : 40 years old
# BW    : 70kg
# Dose: 70 mg on day 1,followed by 50 mg QD,Infusion for 1h

set.seed(123456)

# 2-comp model
mod7 <- RxODE({
  CL     = TVCL*exp(iiv.CL);
  V1     = TVV1*exp(iiv.V1);
  Q      = TVQ;
  V2     = TVV2*exp(iiv.V2);

  C1     = centr/V1;
  C2     = peri/V2;

  d/dt(centr) = - CL*C1 - Q*C1 + Q*C2;
  d/dt(peri)    = Q*C1 - Q*C2;
```

```
  cp = C1*(1 + prop.err.sd)
})

# Adults
# Define typical patient: adults
BW   <- 70   # kg
AGE <- 40     # years old


# Define fixed effect parameters
theta7<- c(TVCL =0.563*(BW/70)^0.75,
           TVV1 =6.04*(BW/70)^1,
           TVV2 =5.13*(BW/70)^1,
           TVQ   =1.31)

# Define between subject variability
omega7 <- lotri(iiv.CL + iiv.V1 ~ C(0.247^2,
                                    0.868   ,0.282^2),
                iiv.V2 ~ 0.494^2)

# Define unexplained variability
sigma7 <- lotri(prop.err.sd ~ 0.199^2)

# DOSE of CAS(iv)
dose_cas_day1 <-70     # mg 1-h infusion
dose_cas_later<-50     # mg 1-h infusion

# Define event record
ev7 <- et(amountUnits="mg", timeUnits="hours") %>%
  add.dosing(dosing.to = "centr",dose = dose_cas_day1, rate = dose_cas_day1/1,
             nbr.doses = 1, start.time = 0) %>%
  add.dosing(dosing.to = "centr",dose = dose_cas_later, rate = dose_cas_later/1,
             nbr.doses = 3, dosing.interval = 24,start.time = 24) %>%
  # sampling:0-24h after the last dosing
  add.sampling(seq(from=72,to=96,by=1))

# Perform simulation
# total number of subject: 1000
sim7   <- rxSolve(mod7,theta7,ev7,omega=omega7,sigma=sigma7,nSub=1000)

# Concentration-Time plot
pk_CAS7 <- sim7 %>%
  group_by(time) %>%
  # 10-90% Prediction interval
```

```
  summarise(medCL = median(CL),
            medconc = median(cp),
            lowconc = quantile(cp,0.1),
            highconc = quantile(cp, 0.9)) %>%
  ungroup() %>%
  # time after last dose
  mutate(time = as.numeric(time),
         tald = time-72)

dat7 <- pk_CAS7 %>%
  mutate(study="Moor et al.(2021)",
         drug="CAS",
         pop="Adult")

# 8----Wu et al.(2022)-----
# Define the 2-comp model,model info

# CL(L/h) =0.385(5%)
# Vc(L)    =4.27(12%)
# Q(L/h)   =2.85(11%)
# Vp(L)    =6.01(13%)

# CL = TVCL* exp(eta.CL) *(ALB/37.42)^(-1.01)
# V1 = TVV1* exp(eta.V1)
# Q   = TVQ * exp(eta.Q)
# V2 = TVV2* exp(eta.V2)

# IIV(CL) =0.335
# IIV(V1) =0.675
# IIV(Q)   =0 FIX
# IIV(V2) =0.477

# error=0.134
# additive error=0.213

# Typical population:
# Adults : Heart transplantation
# age : 40 years old
# BW   : 70kg
# ALB level : 37.42 g/L
# Dose: 70 mg on day 1,followed by 50 mg QD,Infusion for 1h

set.seed(12345)
```

```
# 2-comp model
mod8 <- RxODE({
   CL = TVCL* exp(iiv.CL)
   V1 = TVV1* exp(iiv.V1)
   Q    = TVQ
   V2 = TVV2* exp(iiv.V2)

   C1      = centr/V1;
   C2      = peri/V2;

   d/dt(centr) = - CL*C1 - Q*C1 + Q*C2;
   d/dt(peri)    = Q*C1 - Q*C2;

   cp = C1*(1 + prop.err.sd) + add.err.sd
})

# Define typical patient: adults
BW    <- 70             # kg
ALB <- 37.42       # g/L

# Define fixed effect parameters
theta8<- c(TVCL =0.385*(ALB/37.42)^(-1.01),
                   TVV1 =4.27,
                   TVV2 =6.01,
                   TVQ    =2.85)

# Define between subject variability
omega8 <- lotri(iiv.CL ~ 0.335^2,iiv.V1 ~ 0.675^2 ,iiv.V2 ~ 0.477^2)

# Define unexplained variability
sigma8 <- lotri(prop.err.sd ~ 0.134^2, add.err.sd ~ 0.213)

# DOSE of CAS(iv)
dose_cas_day1 <-70       # mg 1-h infusion
dose_cas_later<-50       # mg 1-h infusion

# Define event record
ev8 <- et(amountUnits="mg", timeUnits="hours") %>%
   add.dosing(dosing.to = "centr",dose = dose_cas_day1, rate = dose_cas_day1/1,
                   nbr.doses = 1, start.time = 0) %>%
   add.dosing(dosing.to = "centr",dose = dose_cas_later, rate = dose_cas_later/1,
                   nbr.doses = 3, dosing.interval = 24,start.time = 24) %>%
   # sampling:0-24h after the last dosing
   add.sampling(seq(from=72,to=96,by=1))
```

```
# Perform simulation
# total number of subject: 1000
sim8    <- rxSolve(mod8,theta8,ev8,omega=omega8,sigma=sigma8,nSub=1000)

# Concentration-Time plot
pk_CAS8 <- sim8 %>%
   group_by(time) %>%
   # 10-90% Prediction interval
   summarise(medCL = median(CL),
                    medconc = median(cp),
                    lowconc = quantile(cp,0.1),
                    highconc = quantile(cp, 0.9)) %>%
   ungroup() %>%
   # time after last dose
   mutate(time = as.numeric(time),
              tald = time-72)

dat8 <- pk_CAS8 %>%
   mutate(study="Wu et al.(2022)",
              drug="CAS",
              pop="Adult")

# 9----Pressiat et al.(2022)-----
# Define the 2-comp model,model info

# CL(L/h) =0.38(8.16%)
# V1(L)      =6.24(15.8%)
# Q(L/h)    =2.58(0.2%)
# V2(L)      =6.44(28.5%)

# IIV(CL) =0.33(18.6%)
# IIV(V1) =0.59(21.6%)
# IIV(V2) =1.07(21.9%)

# prop error=0.36(4.4%)

# Typical population:
# Adults : Liver Transplant Recipients
# age : 40 years old
# BW    : 70kg
# Dose: 70 mg on day 1,followed by 50 mg QD,Infusion for 1h

set.seed(12345)
```

```
rxSetseed(12345)

# 2-comp model
mod9 <- RxODE({
   CL = TVCL* exp(iiv.CL)
   V1 = TVV1* exp(iiv.V1)
   Q    = TVQ
   V2 = TVV2* exp(iiv.V2)

   C1      = centr/V1;
   C2      = peri/V2;

   d/dt(centr) = - CL*C1 - Q*C1 + Q*C2;
   d/dt(peri)    = Q*C1 - Q*C2;

   cp = C1*(1 + prop.err.sd)
})

# Define fixed effect parameters
theta9<- c(TVCL    = 0.38,
                  TVV1    = 6.24,
                  TVV2    = 6.44,
                  TVQ      = 2.58)

# Define between subject variability
omega9 <- lotri(iiv.CL ~ 0.33^2,iiv.V1 ~ 0.59^2 ,iiv.V2 ~ 1.07^2)

# Define unexplained variability
sigma9<- lotri(prop.err.sd ~ 0.36)

# DOSE of CAS(iv)
dose_cas_day1 <-70      # mg 1-h infusion
dose_cas_later<-50      # mg 1-h infusion

# Define event record
ev9 <- et(amountUnits="mg", timeUnits="hours") %>%
   add.dosing(dosing.to = "centr",dose = dose_cas_day1, rate = dose_cas_day1/1,
                    nbr.doses = 1, start.time = 0) %>%
   add.dosing(dosing.to = "centr",dose = dose_cas_later, rate = dose_cas_later/1,
                    nbr.doses = 3, dosing.interval = 24,start.time = 24) %>%
   # sampling:0-24h after the last dosing
   add.sampling(seq(from=72,to=96,by=1))

# Perform simulation
```

```
# total number of subject: 1000
sim9    <- rxSolve(mod9,theta9,ev9,omega=omega9,sigma=sigma9,nSub=1000)

# Concentration-Time plot
pk_CAS9 <- sim9 %>%
   group_by(time) %>%
   # 10-90% Prediction interval
   summarise(medCL = median(CL),
                   medconc = median(cp),
                   lowconc = quantile(cp,0.1),
                   highconc = quantile(cp, 0.9)) %>%
   ungroup() %>%
   # time after last dose
   mutate(time = as.numeric(time),
            tald = time-72)

dat9 <- pk_CAS9 %>%
   mutate(study="Pressiat et al.(2022)",
            drug="CAS",
            pop="Adult")


# 15----Yang et al.(2022)-----
# Define the 1-comp model,model info

# CL(L/h) =0.32*(1+0.46*ALB*)*(1+0.98*WT*)

# V(L)      ==13.31*(1+0.49*ALB*)*(1+0.24*WT*)

# ALB*=1, ALB<35 g/L;
# ALB*=0, ALB≥35 g/L;
# WT*=1, WT≥70 kg,
# WT*=0, WT< 70 kg)

# WT*=1, ALB*=0

# CL(L/h) =0.32*(1+0.98)

# V(L)      ==13.31*(1+0.24)

# IIV(CL) =0.292
# IIV(V1) =0.592

# prop error=19.3
```

```
# Typical population:
# Adultsye
# age : 40 years old
# BW    : 70kg
# Dose: 70 mg on day 1,followed by 50 mg QD,Infusion for 1h

set.seed(12345)
rxSetseed(12345)

# 2-comp model
mod15 <- RxODE({
   CL =TVCL*exp(iiv.CL); #a variable in the model.
   V1 =TVV1*exp(iiv.V1);

   conc=centr/V1;

   d/dt(centr)=-CL*conc;    #central

   cp=conc*(1+prop.err.sd)
})

# Define fixed effect parameters
theta15<- c(TVCL    = 0.6336,
                 TVV1      = 16.5044)

# Define between subject variability
omega15 <- lotri(iiv.CL ~ 0.292^2,iiv.V1 ~ 0.592^2)

# Define unexplained variability
sigma15<- lotri(prop.err.sd ~ 0.193)

# DOSE of CAS(iv)
dose_cas_day1 <-70      # mg 1-h infusion
dose_cas_later<-50      # mg 1-h infusion

# Define event record
ev15 <- et(amountUnits="mg", timeUnits="hours") %>%
   add.dosing(dosing.to = "centr",dose = dose_cas_day1, rate = dose_cas_day1/1,
                 nbr.doses = 1, start.time = 0) %>%
   add.dosing(dosing.to = "centr",dose = dose_cas_later, rate = dose_cas_later/1,
                 nbr.doses = 3, dosing.interval = 24,start.time = 24) %>%
   # sampling:0-24h after the last dosing
   add.sampling(seq(from=72,to=96,by=1))
```

```
# Perform simulation
# total number of subject: 1000
sim15    <- rxSolve(mod15,theta15,ev15,omega=omega15,sigma=sigma15,nSub=1000)

# Concentration-Time plot
pk_CAS15 <- sim15 %>%
   group_by(time) %>%
   # 10-90% Prediction interval
   summarise(medCL = median(CL),
                  medconc = median(cp),
                  lowconc = quantile(cp,0.1),
                  highconc = quantile(cp, 0.9)) %>%
   ungroup() %>%
   # time after last dose
   mutate(time = as.numeric(time),
             tald = time-72)

dat15 <- pk_CAS15 %>%
   mutate(study="Yang et al.(2022)",
             drug="CAS",
             pop="Adult")


#----select data----
dat1 <- dat1 %>%
   select(tald,lowconc,medconc,highconc,study,drug,pop)

dat2 <- dat2 %>%
   select(tald,lowconc,medconc,highconc,study,drug,pop)

dat3 <- dat3 %>%
   select(tald,lowconc,medconc,highconc,study,drug,pop)

dat4 <- dat4 %>%
   select(tald,lowconc,medconc,highconc,study,drug,pop)

dat5 <- dat5 %>%
   select(tald,lowconc,medconc,highconc,study,drug,pop)

dat6 <- dat6 %>%
   select(tald,lowconc,medconc,highconc,study,drug,pop)

dat7 <- dat7 %>%
```

```
  select(tald,lowconc,medconc,highconc,study,drug,pop)

dat8 <- dat8 %>%
  select(tald,lowconc,medconc,highconc,study,drug,pop)

dat9 <- dat9 %>%
  select(tald,lowconc,medconc,highconc,study,drug,pop)

dat15 <- dat15 %>%
  select(tald,lowconc,medconc,highconc,study,drug,pop)
#----combine the plots (adults)----

# combine data
dat_cas_adult <- dat1 %>%
  bind_rows(dat2) %>%
  bind_rows(dat3) %>%
  bind_rows(dat4) %>%
  bind_rows(dat5) %>%
  bind_rows(dat6) %>%
  bind_rows(dat7) %>%
  bind_rows(dat8) %>%
  bind_rows(dat9) %>%
  bind_rows(dat15)

# arrange by levels
dat_cas_adult$study <- factor(dat_cas_adult$study, levels = c("Würthwein et al.(2011)",
                                                              "Würthwein et
al.(2013)",
                                                              "Pérez-Pitarch et al.
(2018)",
                                                              "Wang et al.(2020)",
                                                              "Bailly et al.(2020)",
                                                              "Li et al.(2021)",
                                                              "Moor et al.(2021)",
                                                              "Wu et al.(2022)",
                                                              "Pressiat et
al.(2022)",
                                                              "Yang et al.(2022)"))

# change the label names
labelname1 <-   c(   "Würthwein et al.(2011)" ="Würthwein (2011)" ,
                     "Würthwein et al.(2013)" ="Würthwein (2013)" ,
                     "Pérez-Pitarch et al. (2018)" ="Pérez-Pitarch (2018)" ,
                     "Wang et al.(2020)"       ="Wang (2020)"    ,
```

```
                           "Bailly et al.(2020)"       ="Bailly (2020)" ,
                           "Li et al.(2021)"            = "Li (2021)" ,
                           "Moor et al.(2021)"          = "Moor (2021)",
                           "Wu et al.(2022)"            = "Wu (2022)" ,
                           "Pressiat et al.(2022)"   = "Pressiat (2022)",
                           "Yang et al.(2022)"          = "Yang (2022)")

pl1 <- dat_cas_adult %>%
  mutate(lowconc=lowconc/70,
         medconc=medconc/70,
         highconc=highconc/70)%>%
  ggplot(mapping=aes(x=tald, y=medconc)) +
  geom_line(size=1.5, color="#003B8E") +
  geom_ribbon(aes(x=tald,ymin=lowconc,ymax=highconc), fill = "#003B8E",alpha=0.2) +
  theme_bw(base_size = 30) +
  facet_wrap(~study, ncol = 5,
             labeller = labeller(study=as_labeller(labelname1)),
             scales="free") +
  scale_x_continuous("Time (h)", limits = c(0,24.1), breaks = c(0,4,8,12,16,20,24)) +
  scale_y_continuous("Concentration (mg/L/kg)") +
  coord_cartesian(ylim = c(0,0.4)) +
  ggtitle("c Adults: 70 kg, 40-year-old") +
  theme (legend.position    = "none",
         plot.background    = element_rect(fill = 'white'),
         panel.grid.minor = element_blank(),
         panel.grid.major = element_blank(),
         plot.title = element_text(hjust = 0,
                                   size = 30),
         strip.background = element_rect(fill = "#CCD8E8"))

#export picture
jpeg(filename = paste0(output_dir, "/Caspofungin_Adult_plot2.jpg"), width=9000, height=4000,
res=300)
print(pl1)
dev.off()


dat_cas_adult2 <- dat1 %>%
  bind_rows(dat2) %>%
  bind_rows(dat3) %>%
  bind_rows(dat5) %>%
  bind_rows(dat6) %>%
  bind_rows(dat7) %>%
  bind_rows(dat8) %>%
```

```
  bind_rows(dat9)

# change the label names
labelname4 <-   c(   "Würthwein et al.(2011)" ="Würthwein (2011)" ,
                       "Würthwein et al.(2013)" ="Würthwein (2013)" ,
                       "Pérez-Pitarch et al. (2018)" ="Alejandro (2018)" ,
                       "Bailly et al.(2020)"       ="Bailly (2020)" ,
                       "Li et al.(2021)"            = "Li (2021)" ,
                       "Moor et al.(2021)"          = "Moor (2021)",
                       "Wu et al.(2022)"            = "Wu (2022)" ,
                       "Pressiat et al.(2022)"   ="Pressiat (2022)")



pl2 <- dat_cas_adult2 %>%
  ggplot(mapping=aes(x=tald, y=medconc)) +
  geom_line(size=1.5, color="#003B8E") +
  geom_ribbon(aes(x=tald,ymin=lowconc,ymax=highconc), fill = "#003B8E",alpha=0.2) +
  theme_bw(base_size = 30) +
  facet_wrap(~study, ncol = 4,
             labeller = labeller(study=as_labeller(labelname4)),
             scales="free") +
  scale_x_continuous("Time (h)", limits = c(0,24.1), breaks = c(0,4,8,12,16,20,24)) +
  scale_y_continuous("Concentration (mg/L)") +
  coord_cartesian(ylim = c(0,20)) +
  ggtitle("c Adults: 70 kg, 40-year-old") +
  theme (legend.position   = "none",
         plot.background    = element_rect(fill = 'white'),
         panel.grid.minor = element_blank(),
         panel.grid.major = element_blank(),
         plot.title = element_text(hjust = 0,
                                   size = 30),
         strip.background = element_rect(fill = "#CCD8E8"))

#export picture
jpeg(filename = paste0(output_dir, "/Caspofungin_Adult_plot_ref.jpg"), width=9000, height=4000,
res=300)
print(pl2)
dev.off()

#-----------------------------------------
#-----Children 20KG 6Y 100cm BSA 0.79---------
#-----------------------------------------

# 10----Yang et al.(2019)----
```

```
# Define the 2-comp model,model info

# CL(L/h)    = 0.165
# V1(L)        = 1.730
# Q(L/h)      = 0.351
# V2(L)        = 0.943
# FBSA-CL    = (BSA/0.79)^1.3
# FBSA-V1    = (BSA/0.79)^1.5

# CL = TVCL*(BSA/0.79)^1.3*exp(iiv.CL)
# V1 = TVV1*(BSA/0.79)^1.5
# Q    = TVQ* exp(iiv.Q)
# V2 = TVV2 × exp(iiv.V2)

# IIV(CL) =0.242(21.0%)
# IIV(Q)    =1.616(90.0%)
# IIV(V2) =0.766(71.6%)

# Proportional error=0.196
# BSA,body surface area(m^2)

# Typical population:
# Children Allogeneic Hematopoietic Stem Cell Recipients
# age : 6 years old
# BW    : 20kg
# HT    : 100cm
# BSA : 0.79m^2
# Dose: 70 mg/m^2 on day 1,followed by 50 mg/m^2 QD,Infusion for 1h

set.seed(123456789)

# 2-comp model
mod10 <- RxODE({
   CL      = TVCL*exp(iiv.CL);
   V1      = TVV1;
   Q       = TVQ*exp(iiv.Q);
   V2      = TVV2*exp(iiv.V2);

   C1      = centr/V1;
   C2      = peri/V2;

   d/dt(centr) = - CL*C1 - Q*C1 + Q*C2;
   d/dt(peri)    = Q*C1 - Q*C2;
```

```
  cp = C1*(1 + prop.err.sd)
})

# Adults
# Define typical patient: adults
BSA   <- 0.79      # m^2

# Define fixed effect parameters
theta10<- c(TVCL =0.165*(BSA/0.79)^1.3,
            TVV1 =1.73*(BSA/0.79)^1.5,
            TVQ   =0.351,
            TVV2 =0.943)

# Define between subject variability
omega10 <- lotri(iiv.CL ~ 0.242^2,
                 iiv.Q   ~ 1.616^2,
                 iiv.V2 ~ 0.766^2 )

# Define unexplained variability
sigma10 <- lotri(prop.err.sd ~ 0.196)

# DOSE of CAS(iv)
dose_cas_day1 <-70*BSA      # mg 1-h infusion
dose_cas_later<-50*BSA   # mg 1-h infusion

# Define event record
ev10 <- et(amountUnits="mg", timeUnits="hours") %>%
  add.dosing(dosing.to = "centr",dose = dose_cas_day1, rate = dose_cas_day1/1,
             nbr.doses = 1, start.time = 0) %>%
  add.dosing(dosing.to = "centr",dose = dose_cas_later, rate = dose_cas_later/1,
             nbr.doses = 3, dosing.interval = 24,start.time = 24) %>%
  # sampling:0-24h after the last dosing
  add.sampling(seq(from=72,to=96,by=1))

# Perform simulation
# total number of subject: 1000
sim10   <- rxSolve(mod10,theta10,ev10,omega=omega10,sigma=sigma10,nSub=1000)

# Concentration-Time plot
pk_CAS10 <- sim10 %>%
  group_by(time) %>%
  # 10-90% Prediction interval
  summarise(medCL = median(CL),
            medconc = median(cp),
```

```
                    lowconc = quantile(cp,0.1),
                    highconc = quantile(cp, 0.9)) %>%
  ungroup() %>%
  # time after last dose
  mutate(time = as.numeric(time),
         tald = time-72)

dat10 <- pk_CAS10 %>%
  mutate(study="Yang et al.(2019)",
         drug="CAS",
         pop="Child")

# 11----Niu et al.(2020)------
# Define the 1-comp model,model info

# TVCL(L/h) =0.14(8.50%)
# TVVd(L)     =1.36(11.8 %)

# CL(L/h)     =0.14*(BSA/0.79)^0.89*(InAST/3.38)^(-0.23)
# Vd(L)        =1.36*(BSA/0.79)

# IIV(CL) =33.3%
# IIV(Vd) =32.9%

# prop error=0.266

# Typical population:
# Children With Allogeneic Hematopoietic Stem Cell Transplantation
# age : 6 years old
# BSA : 0.79m^2
# AST : 3.38
# BW    : 20kg
# Dose: 70 mg/m^2 on day 1,followed by 50 mg/m^2 QD,Infusion for 1h

set.seed(1234567898)

# 1-comp model
mod11 <- RxODE({
  CL =TVCL*exp(iiv.CL); #a variable in the model.
  Vd =TVVd*exp(iiv.Vd);

  conc=centr/Vd;

  d/dt(centr)=-CL*conc;   #central
```

```
  cp=conc*(1+prop.err.sd)
})

# Define typical patient:
# Children With Allogeneic Hematopoietic Stem Cell Transplantation
# Age    : 10 years old
# BW      : 30kg
# BSA    : 0.79 m^2
# lnAST: 3.38

BSA    <- 0.79
lnAST<- 3.38

# Define fixed effect parameters
theta11<- c(TVCL =0.14*(BSA/0.79)^0.89*(lnAST/3.38)^(-0.23),
                       TVVd =1.36*(BSA/0.79))

# Define between subject variability
omega11 <- lotri(iiv.CL ~ 0.333^2, iiv.Vd ~ 0.329^2)

# Define unexplained variability
sigma11 <- lotri(prop.err.sd ~ 0.266^2)

# DOSE of CAS(iv)
dose_cas_day1 <-70*BSA    # mg 1-h infusion
dose_cas_later<-50*BSA      # mg 1-h infusion

# Define event record
ev11 <- et(amountUnits="mg", timeUnits="hours") %>%
  add.dosing(dosing.to = "centr",dose = dose_cas_day1, rate = dose_cas_day1/1,
                    nbr.doses = 1, start.time = 0) %>%
  add.dosing(dosing.to = "centr",dose = dose_cas_later, rate = dose_cas_later/1,
                    nbr.doses = 3, dosing.interval = 24,start.time = 24) %>%
  # sampling:0-24h after the last dosing
  add.sampling(seq(from=72,to=96,by=1))

# Perform simulation
# total number of subject: 1000
sim11    <- rxSolve(mod11,theta11,ev11,omega=omega11,sigma=sigma11,nSub=1000)

# Concentration-Time plot
pk_CAS11 <- sim11 %>%
  group_by(time) %>%
```

```
  # 10-90% Prediction interval
  summarise(medCL = median(CL),
            medconc = median(cp),
            lowconc = quantile(cp,0.1),
            highconc = quantile(cp, 0.9)) %>%
  ungroup() %>%
  # time after last dose
  mutate(time = as.numeric(time),
         tald = time-72)

dat11 <- pk_CAS11 %>%
  mutate(study="Niu et al.(2020)",
         drug="CAS",
         pop="Child")

# 12----Gastine et al.(2022)-----
# Define the 2-comp model,model info

# CL(L/h/70kg)   = 0.790
# V1(L/70kg)     = 7.75
# Q(L/h/70kg)    = 1.2
# V2(L/70kg)     = 5.29

# IIV(CL) = 0.275
# IIV(V1) = 0.315
# IIV(V2) = 0.151

# IOV(CL) = 0.172

# Proportional error=0.194

# Typical population:
# Children paediatric patients
# age : 6 years old
# BW   : 20kg
# BSA : 0.79m^2
# Dose: 70 mg/m^2 on day 1,followed by 50 mg/m^2 QD,Infusion for 1h

set.seed(123456789)

# 2-comp model
mod12 <- RxODE({
  CL     = TVCL*exp(iiv.CL);
  V1     = TVV1*exp(iiv.V1);
```

```
    Q       = TVQ;
    V2      = TVV2*exp(iiv.V2);

    C1      = centr/V1;
    C2      = peri/V2;

    d/dt(centr) = - CL*C1 - Q*C1 + Q*C2;
    d/dt(peri)    = Q*C1 - Q*C2;

    cp = C1*(1 + prop.err.sd)
})

# Define typical patient:
# children
# BW :30 kg
BW <- 20
BSA<- 0.79

# Define fixed effect parameters
theta12<- c(TVCL =0.79*(BW/70),
            TVV1 =7.75*(BW/70),
            TVQ    =1.2*(BW/70),
            TVV2 =5.29*(BW/70))

# Define between subject variability
omega12 <- lotri(iiv.CL ~ 0.275^2,
                 iiv.V1 ~ 0.315^2,
                 iiv.V2 ~ 0.151^2)

# Define unexplained variability
sigma12 <- lotri(prop.err.sd ~ 0.194^2)

# DOSE of CAS(iv)
dose_cas_day1 <-70*BSA      # mg 1-h infusion
dose_cas_later<-50*BSA    # mg 1-h infusion

# Define event record
ev12 <- et(amountUnits="mg", timeUnits="hours") %>%
    add.dosing(dosing.to = "centr",dose = dose_cas_day1, rate = dose_cas_day1/1,
               nbr.doses = 1, start.time = 0) %>%
    add.dosing(dosing.to = "centr",dose = dose_cas_later, rate = dose_cas_later/1,
               nbr.doses = 3, dosing.interval = 24,start.time = 24) %>%
    # sampling:0-24h after the last dosing
    add.sampling(seq(from=72,to=96,by=1))
```

```
# Perform simulation
# total number of subject: 1000
sim12    <- rxSolve(mod12,theta12,ev12,omega=omega12,sigma=sigma12,nSub=1000)

# Concentration-Time plot
pk_CAS12 <- sim12 %>%
   group_by(time) %>%
   # 10-90% Prediction interval
   summarise(medCL = median(CL),
                   medconc = median(cp),
                   lowconc = quantile(cp,0.1),
                   highconc = quantile(cp, 0.9)) %>%
   ungroup() %>%
   # time after last dose
   mutate(time = as.numeric(time),
              tald = time-72)

dat12 <- pk_CAS12 %>%
   mutate(study="Gastine et al.(2022)",
              drug="CAS",
              pop="Child")

#----plot_children-----
dat10 <- dat10 %>%
   select(tald,lowconc,medconc,highconc,study,drug,pop)

dat11 <- dat11 %>%
   select(tald,lowconc,medconc,highconc,study,drug,pop)

dat12 <- dat12 %>%
   select(tald,lowconc,medconc,highconc,study,drug,pop)

# combine data
dat_cas_child <- dat10 %>%
   bind_rows(dat11) %>%
   bind_rows(dat12)

# arrange by levels
dat_cas_child$study <- factor(dat_cas_child$study, levels = c("Yang et al.(2019)",
                                                              "Niu et al.(2020)",
                                                              "Gastine          et
al.(2022)"))
```

```
# change the label names
labelname2 <-    c(    "Yang et al.(2019)"        = "Yang(2019)",
                        "Niu et al.(2020)"         = "Niu(2020)",
                        "Gastine et al.(2022)" = "Gastine(2022)")

pl3 <- dat_cas_child %>%
  mutate(lowconc=lowconc/20,
         medconc=medconc/20,
         highconc=highconc/20)%>%
  ggplot(mapping=aes(x=tald, y=medconc)) +
  geom_line(size=1.5, color="#003B8E") +
  geom_ribbon(aes(x=tald,ymin=lowconc,ymax=highconc), fill = "#003B8E",alpha=0.2) +
  theme_bw(base_size = 30) +
  facet_wrap(~study, ncol = 3,
             labeller = labeller(study=as_labeller(labelname2)),
             scales="free") +
  scale_x_continuous("Time (h)", limits = c(0,24.1), breaks = c(0,4,8,12,16,20,24)) +
  scale_y_continuous("Concentration (mg/L/kg)") +
  coord_cartesian(ylim = c(0,2.5)) +
  ggtitle("b Children: 110cm,20 kg,6-year-old") +
  theme (legend.position    = "none",
         plot.background    = element_rect(fill = 'white'),
         panel.grid.minor = element_blank(),
         panel.grid.major = element_blank(),
         plot.title = element_text(hjust = 0,
                                   size = 30),
         strip.background = element_rect(fill = "#CCD8E8"))
#export picture
jpeg(filename = paste0(output_dir, "/Caspofungin_Child_plot_ref2.jpg"), width=9000,
height=4000, res=300)
print(pl3)
dev.off()

#----------------------------------------
#-----Infants 10KG 1Y--------------------
#----------------------------------------
# 13----Niu et al.(2020)------
# Define the 1-comp model,model info

# TVCL(L/h) =0.14(8.50%)
# TVVd(L)      =1.36(11.8 %)

# CL(L/h)      =0.14*(BSA/0.79)^0.89*(InAST/3.38)^（-0.23）
# Vd(L)          =1.36*(BSA/0.79)
```

```
# IIV(CL) =33.3%
# IIV(Vd) =32.9%

# prop error=0.266

# Typical population:
# Infants With Allogeneic Hematopoietic Stem Cell Transplantation
# age : 1 years old
# BSA = (WT*HT/3600)^0.5
# BSA : 0.441 m^2
# BW    : 10kg
# Height: 70cm
# Dose: 70 mg/m^2 on day 1,followed by 50 mg/m^2 QD,Infusion for 1h
set.seed(1234567898)

# 1-comp model
mod13 <- RxODE({
   CL =TVCL*exp(iiv.CL); #a variable in the model.
   Vd =TVVd*exp(iiv.Vd);

   conc=centr/Vd;

   d/dt(centr)=-CL*conc;#central

   cp=conc*(1+prop.err.sd)#RUV
})

# Adults
# Define typical patient:
# Children With Allogeneic Hematopoietic Stem Cell Transplantation
# Age    : 10 years old
# BW      : 30kg
# BSA    : 0.79 m^2
# InAST: 3.38

BSA    <- 0.441
InAST<- 3.38

# Define fixed effect parameters
theta13<- c(TVCL =0.14*(BSA/0.79)^0.89*(InAST/3.38)^(-0.23),
                    TVVd =1.36*(BSA/0.79))

# Define between subject variability
```

```
omega13 <- lotri(iiv.CL ~ 0.333^2, iiv.Vd ~ 0.329^2)

# Define unexplained variability
sigma13 <- lotri(prop.err.sd ~ 0.266^2)

# DOSE of CAS(iv)
dose_cas_day1 <-70*BSA    # mg 1-h infusion
dose_cas_later<-50*BSA      # mg 1-h infusion

# Define event record
ev13 <- et(amountUnits="mg", timeUnits="hours") %>%
   add.dosing(dosing.to = "centr",dose = dose_cas_day1, rate = dose_cas_day1/1,
                      nbr.doses = 1, start.time = 0) %>%
   add.dosing(dosing.to = "centr",dose = dose_cas_later, rate = dose_cas_later/1,
                      nbr.doses = 3, dosing.interval = 24,start.time = 24) %>%
   # sampling:0-24h after the last dosing
   add.sampling(seq(from=72,to=96,by=1))

# Perform simulation
# total number of subject: 1000
sim13    <- rxSolve(mod13,theta13,ev13,omega=omega13,sigma=sigma13,nSub=1000)

# Concentration-Time plot
pk_CAS13 <- sim13 %>%
   group_by(time) %>%
   # 10-90% Prediction interval
   summarise(medCL = median(CL),
                     medconc = median(cp),
                     lowconc = quantile(cp,0.1),
                     highconc = quantile(cp, 0.9)) %>%
   ungroup() %>%
   # time after last dose
   mutate(time = as.numeric(time),
              tald = time-72)

dat13 <- pk_CAS13 %>%
   mutate(study="Niu et al.(2020)",
              drug="CAS",
              pop="Infant")

# 14----Gastine et al.(2022)-----
# Define the 2-comp model,model info

# CL(L/h/70kg)    = 0.790
```

```
# V1(L/70kg)        = 7.75
# Q(L/h/70kg)      = 1.2
# V2(L/70kg)        = 5.29

# IIV(CL) = 0.275(12.0%)
# IIV(V1) = 0.315(21.7%)
# IIV(V2) = 0.151(70.3%)

# Proportional error=0.194

# Typical population:
# Children paediatric patients
# age : 1 years old
# BW    : 10kg
# BSA : 0.441m^2
# Dose: 70 mg/m^2 on day 1,followed by 50 mg/m^2 QD,Infusion for 1h

set.seed(123456789)

# 2-comp model
mod14 <- RxODE({
   CL      = TVCL*exp(iiv.CL );
   V1      = TVV1*exp(iiv.V1);
   Q       = TVQ;
   V2      = TVV2*exp(iiv.V2);

   C1      = centr/V1;
   C2      = peri/V2;

   d/dt(centr) = - CL*C1 - Q*C1 + Q*C2;
   d/dt(peri)    = Q*C1 - Q*C2;

   cp = C1*(1 + prop.err.sd)
})

# Define typical patient:
# children
# BW :30 kg
BW <- 10
BSA<- 0.441

# Define fixed effect parameters
theta14<- c(TVCL =0.79*BW/70,
                     TVV1 =7.75*BW/70,
```

```
                    TVQ    =1.2*BW/70,
                    TVV2 =5.29*BW/70)

# Define between subject variability
omega14 <- lotri(iiv.CL ~ 0.275^2,
                     iiv.V1 ~ 0.315^2,
                     iiv.V2 ~ 0.151^2)

# Define unexplained variability
sigma14 <- lotri(prop.err.sd ~ 0.194^2)

# DOSE of CAS(iv)
dose_cas_day1 <-70*BSA      # mg 1-h infusion
dose_cas_later<-50*BSA    # mg 1-h infusion

# Define event record
ev14 <- et(amountUnits="mg", timeUnits="hours") %>%
   add.dosing(dosing.to = "centr",dose = dose_cas_day1, rate = dose_cas_day1/1,
                  nbr.doses = 1, start.time = 0) %>%
   add.dosing(dosing.to = "centr",dose = dose_cas_later, rate = dose_cas_later/1,
                  nbr.doses = 3, dosing.interval = 24,start.time = 24) %>%
   # sampling:0-24h after the last dosing
   add.sampling(seq(from=72,to=96,by=1))

# Perform simulation
# total number of subject: 1000
sim14    <- rxSolve(mod14,theta14,ev14,omega=omega14,sigma=sigma14,nSub=1000)
```
